# Assembly and comparative analysis of the complete mitochondrial genome of *Brassica rapa* var. *Purpuraria*

**DOI:** 10.1186/s12864-024-10457-1

**Published:** 2024-06-01

**Authors:** Yihui Gong, Xin Xie, Guihua Zhou, Meiyu Chen, Zhiyin Chen, Peng Li, Hua Huang

**Affiliations:** 1 Development and Utilization and Quality and Safety Control of Characteristic Agricultural Resources in Central Hunan, College of Agriculture and Biotechnology , Hunan University of Humanities, Science and Technology, Loudi, 417000 China; 2 Institute of Fruit Tree Research, Key Laboratory of South Subtropical Fruit Biology and Genetic Resource Utilization, Ministry of Agriculture and Rural Affairs, Guangdong Provincial Key Laboratory of Tropical and Subtropical Fruit Tree Research, Guangdong Academy of Agricultural Sciences, Guangzhou, 510640 China; 3 Xiangtan Agricultural Science Research Institute, Xiangtan, 411100 China

**Keywords:** *Brassica rapa* var. *Purpuraria*, Mitochondrial genome, Repeat sequence, RNA editing, Phylogenetic analysis

## Abstract

**Background:**

Purple flowering stalk (*Brassica rapa* var. *purpuraria*) is a widely cultivated plant with high nutritional and medicinal value and exhibiting strong adaptability during growing. Mitochondrial (mt) play important role in plant cells for energy production, developing with an independent genetic system. Therefore, it is meaningful to assemble and annotate the functions for the mt genome of plants independently. Though there have been several reports referring the mt genome of in *Brassica* species, the genome of mt in *B. rapa* var. *purpuraria* and its functional gene variations when compared to its closely related species has not yet been addressed.

**Results:**

The mt genome of *B. rapa* var. *purpuraria* was assembled through the Illumina and Nanopore sequencing platforms, which revealed a length of 219,775 bp with a typical circular structure. The base composition of the whole *B. rapa* var. *purpuraria* mt genome revealed A (27.45%), T (27.31%), C (22.91%), and G (22.32%). 59 functional genes, composing of 33 protein-coding genes (PCGs), 23 tRNA genes, and 3 rRNA genes, were annotated. The sequence repeats, codon usage, RNA editing, nucleotide diversity and gene transfer between the cp genome and mt genome were examined in the *B. rapa* var. *purpuraria* mt genome. Phylogenetic analysis show that *B. rapa* var. *Purpuraria* was closely related to *B. rapa subsp. Oleifera* and *B. juncea*. Ka/Ks analysis reflected that most of the PCGs in the *B. rapa* var. *Purpuraria* were negatively selected, illustrating that those mt genes were conserved during evolution.

**Conclusions:**

The results of our findings provide valuable information on the *B.rapa* var. *Purpuraria* genome, which might facilitate molecular breeding, genetic variation and evolutionary researches for *Brassica* species in the future.

**Supplementary Information:**

The online version contains supplementary material available at 10.1186/s12864-024-10457-1.

## Introduction

Purple flowering stalks (*Brassica rapa* var. *purpuraria*) is widely distributed in the middle regions of the Yangtze River that belongs to the Cruciferae family [1]. *B. rapa* var. *Purpuraria* is an important and popular vegetable for consumers due to its bright color and delicious taste with abundant anthocyanidins, carotenoids, proanthocyanidins, vitamin C and mineral elements [2, 3]. Mitochondria (mt) is important organelles that involve in many metabolic processes related to the synthesis of these nutritional components of amino acids, lipids and vitamins and energy production [4–6]. The mt genome of angiosperm *Arabidopsis thaliana* was first reported in 1997 [7]. Subsequently, the mt genomes of some field crops and fruits, including rice (*Oryza sativa* L.) [8], rape (*Brassica napus* L.) [9], corn (*Zea mays* L.) [10], grape (*Vitis vinifera*)[11], apple (*Malus domestica*) [12], and kiwifruit (*Actinidia chinensis*) [13], have been successively published. The mt genomes have the characteristics of integrity, polymorphism, and semi-autonomy with a unique expression system, they contain a few genes and limited the types of proteins. Therefore, they need to be coordinated with nuclear genes to maintain normal biological functions [14]. The number, arrangement and composition of genes are conserved in the chloroplast (cp) genomes of higher plants [15]. However, Mt genomes have highly conserved characteristics and evolutionary rates that are different from nuclear genes. Therefore, the mt genome is relatively large, which could provide a large amount of genetic information and solve the problem of classification, identification and evolution of related species [14, 16].

The mt genome has significant differences in length, gene sequence, and gene content in different species, and even varies in different cultivars in the same species [17–19]. The length of mt genome in plants varies from 66 Kb to 11.7 Mb [18, 20]. The plant mt genomes mainly contain 50–60 conserved genes, which involved in oxidative phosphorylation and protein translation, and many unknown function open reading frames (ORFs) [21]. In addition, Some of ORFs in the mt genome play a key role in cytoplasmic male sterility of species [22–24]. Except for the unknown function ORFs, the differences in the number of complex II subunit, ribosomal protein genes, tRNAs and multi-copy genes were the main reason for the difference in the number of mt genes in different species [25]. With the development of sequencing technology and the decreasing of sequencing costs, a variety of mt genomes have been reported. The mt genome of Brassica species including *A. thaliana* [26], *Raphanus sativus* L. [27], and *B. napus* [28] have been reported. In addition, the mt genomes of several varieties of *B. rapa* species have also been addressed. The mt genomes in the varieties of B. rapa developed with close full length from 219,736 bp to 219,775 bp, with same number of tRNA (18) and rRNA (3); and exhibited minor differences for gene numbers between 54 and 99, and PCGs ranged from 34 to 78 [29–31]. The characterized mt genome could help to observe structural variations in the evolutionary history of *Brassica* species or varieties. Therefore, assembling and analyzing the mt genomes is important to better understanding of their genetic characteristics and for molecular breeding research.

In this work, the whole mt genome of *B. rapa* var. *Purpuraria* was sequenced and assembled using the Illumina and Nanopore sequencing platforms. The genomic features, repeat sequences, codon usage, RNA editing sites, and comparative genomics were analyzed. We also conducted phylogenetic analysis to understand the genetic variations in *B. rapa* var. *Purpuraria* more effectively. This study enhances our understanding of *B. rapa* var. *Purpuraria* genetics and provides useful information for future researches on identification, molecular breeding and system evolution of mt genomes of Brassicaceae species.

## Materials and methods

### Plant materials, DNA extraction and sequencing

The *B. rapa var. Purpuraria* seeds were provided by Peng Li (Xiangtan Agricultural Science Research Institute), and cultivated at the Xiangtan Agricultural Science Research Institute (Yuhu District, Xiangtan, Hunan, China; 27°52 N, 112°50E) under natural conditions. Fresh young leaves were collected and quickly frozen in liquid nitrogen, and then stored at 80 °C. Plant specimens were conserved in the Hunan University of Humanities, Science and Technology (accession number: 20231218BRP02). The total DNA isolation from the young leaves was performed using CTAB method [32] and purified using Plant DNA Mini Kit (D311, Genepioneer Biotechnologies, Nanjing, China) according to the manufacturer's protocol. The qualified DNA samples was sequenced with 500 bp paired-end (PE) library construction using the VAHTS Universal DNA Library Prep Kit for IlluminaV3 (Vazyme Biotech Co., Ltd, Nanjing, China). About 29,356,547,400 raw data from *B. rapa* var. *Purpuraria* were generated with PE150 sequencing strategy. Subsequently, the qualified DNA was cut into 20-kb fragments using a Covarisg-tube (Covaris) and purified with AMPure beads. The samples were sequenced with Oxford Nanopore library construction.

### Mitochondrial genome assembly and annotation

We used the Fastp 0.23.4 ( https://github.com/OpenGene/fastp) software to filter the second-generation raw data. The parameters were set as follows: (1) Cut off the primer and adapters sequences; (2) Filter out the reads with average quality value lower than Q5. (3) Delete the reads with the number of uncertain bases more than 5. Then, the original tri-generational data was filtered using filtlongv0.2.1 (https://link.zhihu.com/?target=https%3A//github.com/rrwick/Filtlong) with parameters set as:–min_length 1000 and –min_mean_q7. The highly quality tri-generational data were aligned with the plant mt gene database (https://github.com/xul962464/plant_mt_ref_gene) using minimap2 [33]. The size of sequence more than 50 bp, containing multiple core genes and higher alignment quality was selected as the seed sequence. Then, the original tri-generational data were compared with the seed sequences using minimap2, and the sequences with overlap more than 1 kb were screened and added to the seed sequences. The original data were compared to the seed sequence iteratively, and all the third-generation sequencing data of the mt genome were obtained. All the third-generation sequencing data were performed self-correction and assembled using the canu v2.0 program [34], and Bowtie2 (v2.3.5.1) was used to align the second-generation data to the corrected sequence. The second-generation data were compared to stitch the corrected third-generation data using Unicycler (v0.4.8) software with default parameters, followed by using Bandage ( v0.8.1) software to visualize and manually adjust the stitching results. The corrected third-generation sequencing data were aligned to the conting obtained by the second step of Unicycler using minimap2, and the branch direction was manually determined to obtain the final assembly result.

The encoded proteins and rRNAs were aligned to reported plant mt sequences using BLAST, and further manually adjusted according to closely related species. The tRNAs were annotated using tRNAscanSE online tool [35] (http://lowelab.ucsc.edu/tRNAscan-SE/). ORFs were annotated using Open Reading Frame Finder (http://www.ncbi.nlm.nih.gov/gorf/gorf.html) with a minimum length of 102 bp. The map of *B. rapa* var. *Purpuraria* mt genome was drawn using OGDRAW (https://chlorobox.mpimp-golm.mpg.de/OGDraw.html) software. A single nucleotide polymorphism (SNP) was detected among six *Brassica* mt genomes using MUMmer and BLAT v35 softwares.

### Relative synonymous codon usage (RSCU) analysis.

The codon composition of the mt genome of *B. rapa* var. *Purpuraria* was analyzed using a Perl script written by ourselves, to select for a unique coding sequences (CDS) and calculate the RSCU of synonymous codons.

### Analysis of repeat sequences

Dispersed repeat sequences, including forward repeats, backward repeats, reverse repeats, and complementary repeats, were detected using blastn v2.10.1 software with parameters set as -word _ size 7 and evalue 1e-5. Tandem repeats were identified using trf409.linux64 software with parameter set as: 2 7 7 80 10 50 2000 -f -d -m. SSRs were identified with the MISA v1.0 tool [36]. The motif length of one- to six- nucleotide SSRs was set as 10, 5, 4, 3, 3 and 3, respectively.

### Identification of RNA editing sites

RNA editing sites in the PCGs of *B. rapa* var. *Purpuraria* were analyzed using the PmtREP program (http://cloud.genepioneer.com:9929/#/tool/alltool/detail/336) with the cutoff value set as 0.2 [37].

### Gene transfer between the cp genome and mt genome

The *B. rapa* var. *Purpuraria* mt genome was aligned with its cp genome (PP191173) by blast and the selected parameters were set as the matching rate ≥ 70%, E-value ≤ 1e^−5^ and length ≥ 30 bp [38].

### Ka/Ks and Pi analysis

We analyzed the nonsynonymous (Ka) and synonymous (Ks) substitution rates of each PCG between *B. rapa* var. *Purpuraria* and *Cucurbita pepo* (NC_014050.1), *Helianthus grosseserratus* (NC_051989.1), *Brassica oleracea* (NC_016118.1), *Brassica juncea* (NC_016123.1), *Glycyrrhiza uralensis* ( NC_053919) and *Solanum lycopersicum* (MF034192). Homologous gene pairs were aligned in mafft v7.310 ( https://mafft.cbrc.jp/alignment/software/). Ka, Ks, and Ka/Ks values of each PCG were calculated using KaKs_Calculator v2.0 tool (https://sourceforge.net/projects/kakscalculator2/) with MLWL calculation method. Dnasp5 software was used to calculate the Pi value of each gene.

### Phylogenetic analysis

A total of 25 whole mt genomes were used to make sure the phylogenetic position of *B. rapa* var. *Purpuraria*. The 31 mt PCG genes (*atp1*, *atp4*, *atp6*, *atp8*, *atp9*, *ccmB*, *ccmC*, *ccmFc*, *ccmFn*, *cob*, *cox1*, *cox2*, *cox3*, *mttB*, *nad1*, *nad2*, *nad3*, *nad4*, *nad4L*, *nad5*, *nad6*, *nad7*, *nad9*, *rpl16*, *rpl2*, *rpl5*, *rps12*, *rps14*, *rps3*, *rps4*, and *rps7*) conserved across the 25 analyzed species were aligned using Mafft v7.427 software with default parameters. Alignments were trimmed in trimAl with substitution model selecting in ModelFinder [39, 40]. Subsequently, a maximum likelihood tree was constructed by IQ-TREE v1.6.12 software using the mtMet + F + R5 model with a bootstrap of 1000 [41]. *Ginkgo biloba* (NC_027976) was used as the outgroup in this analysis.

## Results

### Characteristics of the *B. rapa* var.* Purpuraria* mt genome

**Fig. 1:**
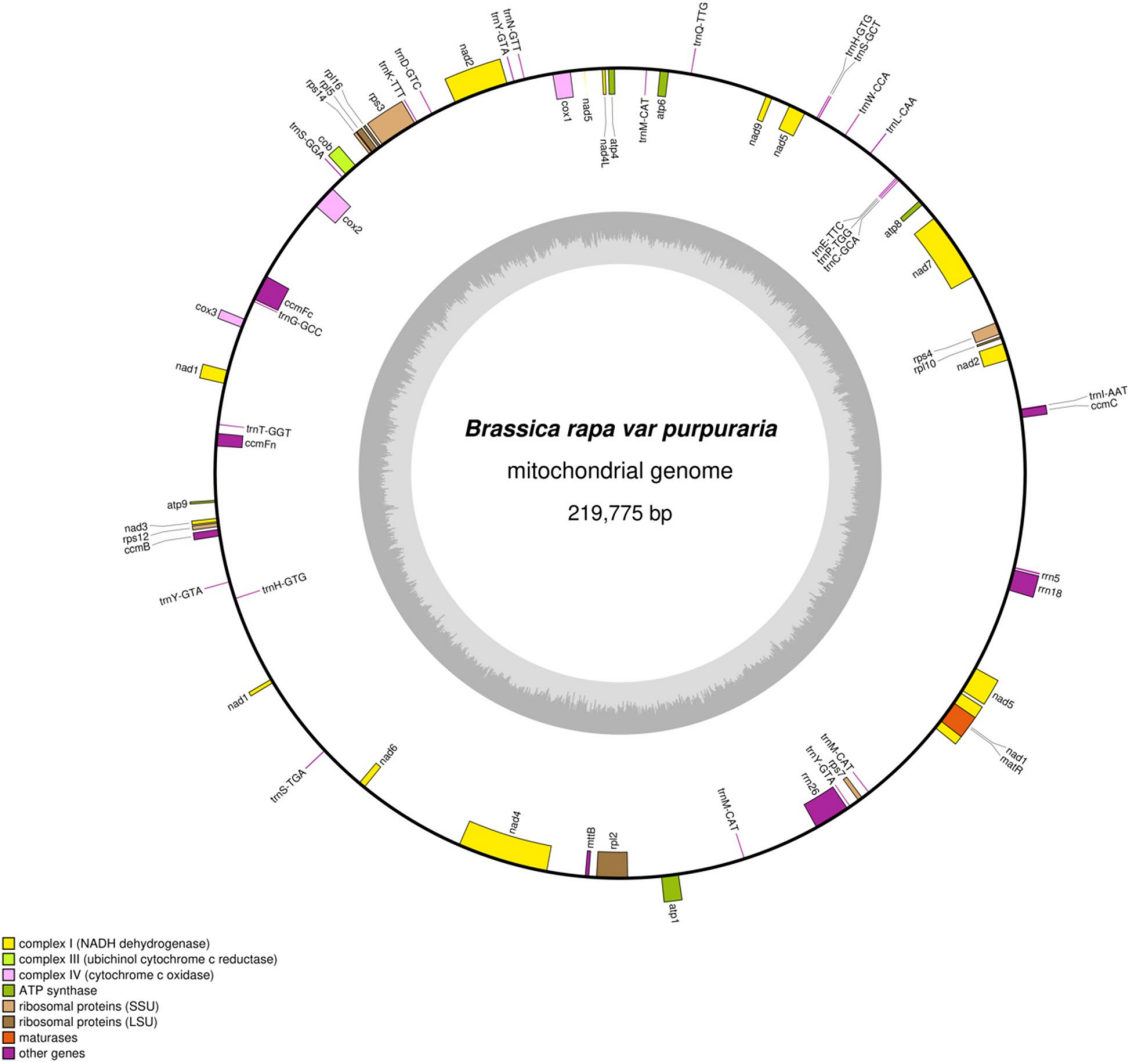
The circular map of the *B. rapa* var. *Purpuraria* mt genome

In this study, the mt genome of *B. rapa* var. *Purpuraria* was sequenced 29,356,547,400 raw data and 97,855,158 bp clean data (Q20 = 97.11% and Q30 = 91.81%) were obtained using the Illumina platform (Table S1). Regarding the Nanopore sequencing, a total of 17,960,842,101 bases and 1,417,067 reads were obtained using the Nanopore sequencing platform. The subreads with N50 and the mean read were 27,413 bp and 12,674 bp, respectively (Table S2). The whole mt genome of *B. rapa* var. *Purpuraria* was 219,775 bp in length with a typical circular structure (Fig. 1). The nucleotide composition of the whole *B. rapa* var. *Purpuraria* mt genome contains 27.45% of A, 27.31% of T, 22.91% of C, and 22.32% of G, with GC and AT contents accounted for 45.23% and 54.77%, respectively (Table S3). PCGs and cis-spliced introns occupied 13.22% and 12.86% of the complete mt genome, whereas tRNA and rRNA genes only made up 0.79% and 2.34%, respectively. A total of 59 genes, comprising 33 PCGs, 3 rRNAs, and 23 tRNAs, were found in the *B. rapa* var. *Purpuraria* mt genome (Table 2). Six genes, namely, *ccmFc*, *cox2*, *rpl2*, *rps3*, *trnI-AAT*, and *trnT-GGT* included one intron, genes of *nad1*, *nad2*, *nad5*, and *nad7* contained four introns, and one gene of *nad4* had three introns. Three tRNA genes were identified in two or three copies (*trnH-GTG*, *trnM-CAT* and *trnY-GTA*) (Fig. 1 and Table 1).

**Table 1:** Gene profile and organization of the *B. rapa* var. *Purpuraria* mt genome

Group of genes	Gene name	Length	Start codon	Stop codon	Amino acid
ATP synthase	*atp1*	1524	ATG	TAG	508
	*atp4*	579	ATG	TAA	193
	*atp6*	786	ATG	TAA	262
	*atp8*	477	ATG	TGA	159
	*atp9*	225	ATG	TGA	75
Cytohrome c biogenesis	*ccmB*	621	ATG	TAA	207
	*ccmC*	744	ATG	TGA	248
	*ccmFc *^*a*^	1329	ATG	CGA(TGA)	443
	*ccmFn*	1146	ATG	TGA	382
Ubichinol cytochrome c reductase	*cob*	1182	ATG	TGA	394
Cytochrome c oxidase	*cox1*	1584	ATG	TAA	528
	*cox2*^*a*^	783	ATG	TAA	261
	*cox3*	798	ATG	TGA	266
Maturases	*matR*	1974	ATG	TAG	658
Transport membrance protein	*mttB*	360	ATG	TAG	120
NADH dehydrogenase	nad1****	978	ACG(ATG)	TAA	326
	nad2****	1467	ATG	TAA	489
	*nad3*	357	ATG	TAA	119
	nad4***	1488	ATG	TGA	496
	*nad4L*	303	ATG	TAA	101
	nad5****	2010	ATG	TAA	670
	*nad6*	618	ATG	TAA	206
	nad7****	1185	ATG	TAG	395
	*nad9*	573	ATG	TAA	191
Ribosomal proteins (LSU)	*rpl10*	225	ATG	TAG	75
	*rpl16*	249	ATG	TAA	83
	*rpl2*^*a*^	1050	ATG	TGA	350
	*rpl5*	558	ATG	TAA	186
Ribosomal proteins (SSU)	*rps12*	378	ATG	TGA	126
	*rps14*	303	ATG	TAG	101
	*rps3*^*a*^	1665	ATG	TAG	555
	*rps4*	1089	ATG	TAA	363
	*rps7*	447	ATG	TAA	149
Succinate dehydrogenase					
Ribosomal RNAs	*rrn18*	1847			
	*rrn26*	3176			
	*rrn5*	121			
Transfer RNAs	*trnC-GCA*	71			
	*trnD-GTC*	74			
	*trnE-TTC*	72			
	*trnG-GCC*	72			
	*trnH-GTG*	74			
	*trnH-GTG*	74			
	*trnI-AAT*^*a*^	69			
	*trnK-TTT*	73			
	*trnL-CAA*	85			
	*trnM-CAT*	73			
	*trnM-CAT*	74			
	*trnM-CAT*	74			
	*trnN-GTT*	72			
	*trnP-TGG*	75			
	*trnQ-TTG*	72			
	*trnS-GCT*	88			
	*trnS-GGA*	87			
	*trnS-TGA*	87			
	*trnT-GGT*^*a*^	69			
	*trnW-CCA*	74			
	*trnY-GTA*	83			
	*trnY-GTA*	68			
	*trnY-GTA*	68			

^a^Intron number

Plant mt genomes have significantly different in size, gene order and content [42]. We selected six *Brassica* mt genomes to compare genome characteristics and determine variability of the mt genome of *B. rapa* var. *Purpuraria* (Table 2)*.* The size of selected mt genomes varied from 219,736 bp (*B. rapa ssp. rapa*) to 232,145 bp (*B. nigra*). The smallest number of genes (53) was found in *B. nigra*, and the largest (106) in *B. napus*. The number of tRNA genes ranged from 17 in *B. napus*, *B. nigra*, and *B. oleracea* to 23 in *B. rapa* var. *Purpuraria*. In addition, all the selected mt genomes included 3 rRNA genes (Table 2). Combing our present results, it revealed that *B. rapa* var. *Purpuraria* has a high degree of similarity to the mt genome sequences to *B. rapa*, *B. nigra*, and *B. oleracea*. Furthermore, to identify sequence variations in the known genes, SNPs were detected between *B. rapa* var. *Purpuraria* and *B. juncea, B. napus, B. rapa, B. nigra* and *B. oleracea.* A total of 202 SNPs were found among six mt genomes. 135 SNPs were identified between *B. rapa* var. *Purpuraria* and *B. rapa ssp. rapa,* followed by 62 SNPs in *B. rapa* var. *Purpuraria* vs *B. nigra* group, 4 SNPs (*cox2*, *atp1*, and two *cox1* genes) in *B. rapa* var. *Purpuraria* vs *B. napus* group, and only one SNP (*atp1* gene) in *B. rapa* var. *Purpuraria* vs *B. oleracea* group. But there were no SNPs identified in *B. rapa* var. *Purpuraria* vs *B. juncea* and *B. rapa* var. *Purpuraria* vs *B. rapa* (Table S4)*.*

**Table 2:** Comparison of gene content among *Brassica* mt genomes

GenBank Accession number	Species	Genome size (bp)	Genes	PCGs	tRNA	rRNA
PP231953	*B. rapa* var. *Purpuraria*	219,775	59	33	23	3
JF920288	*B. juncea*	219,766	99	78	18	3
AP006444.1	*B. napus*	221,853	106	79	17	3
AP017996	*B. rapa*	219,775	55	34	18	3
AP012989	*B. nigra*	232,145	53	33	17	3
AP012988	*B. oleracea*	219,952	54	34	17	3
MT409179	*B. rapa ssp. rapa*	219,736	99	78	18	3

### Codon preference analysis of PCGs

**Fig. 2:**
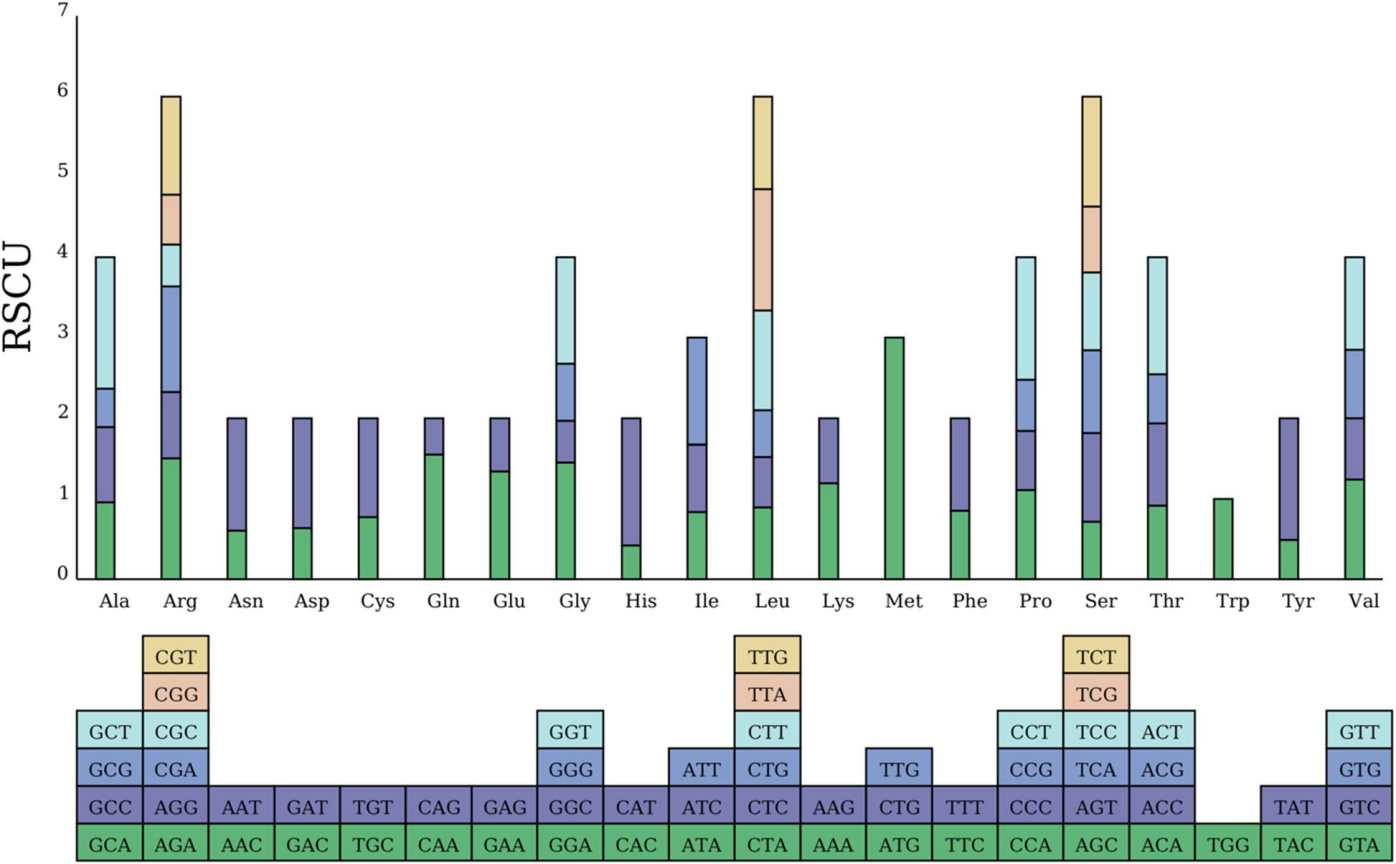
RSCU analysis of the *B. rapa* var.* Purpuraria* mt genome

The total size of PCGs in *B. rapa* var. *Purpuraria* was 35,927 bp. Except for *nad1* gene with ACG as the start codon, ATG was the start codon for other PCGs, which might be the result of C-to-U RNA editing of the second site (Table 1). Four types of stop codons, including TAA, TGA, TAG, and CGA, were detected, and the C to U for RNA editing phenomenon was discovered in *ccmFc* gene. We also calculated the RSCU of 33 PCGs in the *B. rapa* var. *Purpuraria* mt genome (Fig. 2). The 33 PCGs made up 29,055 bp encoding 9685 codons including termination codons. Leucine (Leu) was the most frequent amino acid, with a total of 1053 codons, accounted for 10.87%, followed by serine (Ser), with a total of 856 codons, accounting for 8.84%, and termination codon (Ter) was the rarest with a total of 33 codons, accounting for 0.034%. We discovered that 30 codons of RSCU value were greater than 1, of which 27 codons (90%) ended with A or U, two codons (6.67%) ended with G, and only one codon (3.33%) ended with C. It illustrated that the A / U preference at the third codon was positioned in the *B. rapa* var. *Purpuraria* mt genome (Table S5).

### The prediction of RNA editing

RNA-editing sites are widely distributed in the mt genome of plants. In this study, 379 RNA editing sites within 33 PCGs (Table 3) were predicted in the mt genome of *B. rapa* var. *Purpuraria* using PmtREP tool (Figure S1). Among these PCGs, *atp6* had one RNA-editing site, whereas the highest was in *nad4* with 379 RNA-editing sites (33), of which 30.34% (115 sites) occurred with the first position of the triplet codes, 68.07% (258 sites) located at the second base of the triplet codes. In addition, the first and second bases of the triplet codes were edited, leading to an amino acid change from proline (CCC) to phenylalanine (TTC). There were 45.64% (173 positions) of amino acids hydrophobicity remained unchanged after the RNA editing. Besides, 45.38% (172 positions) of the amino acids were varied from hydrophilic to hydrophobic, while 8.71% (33 positions) were ranged from hydrophobic to hydrophilic. Furthermore, only one amino acid was varied from arginine to stop codon (Table 3). The findings in our study showed that most amino acids were changed from serine to leucine (24.01%, 160 sites), proline to leucine (22.69%, 86 sites), and serine to phenylalanine (12.40%, 47 sites).

**Table 3:** Prediction of RNA editing sites in the *B. rapa* var. *Purpuraria* mt genome

Type	RNA-editing	Number	Percentage
hydrophilic-hydrophilic	CAC (H) = > TAC (Y)	6	12.66%
	CAT (H) = > TAT (Y)	16	
	CGC (R) = > TGC (C)	6	
	CGT (R) = > TGT (C)	20	
hydrophilic-hydrophobic	ACA (T) = > ATA (I)	2	45.38%
	ACC (T) = > ATC (I)	1	
	ACG (T) = > ATG (M)	6	
	ACT (T) = > ATT (I)	4	
	CGG (R) = > TGG (W)	21	
	TCA (S) = > TTA (L)	53	
	TCC (S) = > TTC (F)	16	
	TCG (S) = > TTG (L)	38	
	TCT (S) = > TTT (F)	31	
hydrophilic-stop	CGA (R) = > TGA (X)	1	0.26%
hydrophobic-hydrophilic	CCA (P) = > TCA (S)	6	8.71%
	CCC (P) = > TCC (S)	5	
	CCG (P) = > TCG (S)	5	
	CCT (P) = > TCT (S)	17	
hydrophobic-hydrophobic	CCA (P) = > CTA (L)	34	32.98%
	CCC (P) = > CTC (L)	7	
	CCC (P) = > TTC (F)	6	
	CCG (P) = > CTG (L)	21	
	CCT (P) = > CTT (L)	24	
	CCT (P) = > TTT (F)	10	
	CTC (L) = > TTC (F)	4	
	CTT (L) = > TTT (F)	8	
	GCA (A) = > GTA (V)	2	
	GCC (A) = > GTC (V)	4	
	GCG (A) = > GTG (V)	4	
	GCT (A) = > GTT (V)	1	
Total		379	100%

**Fig. 3:**
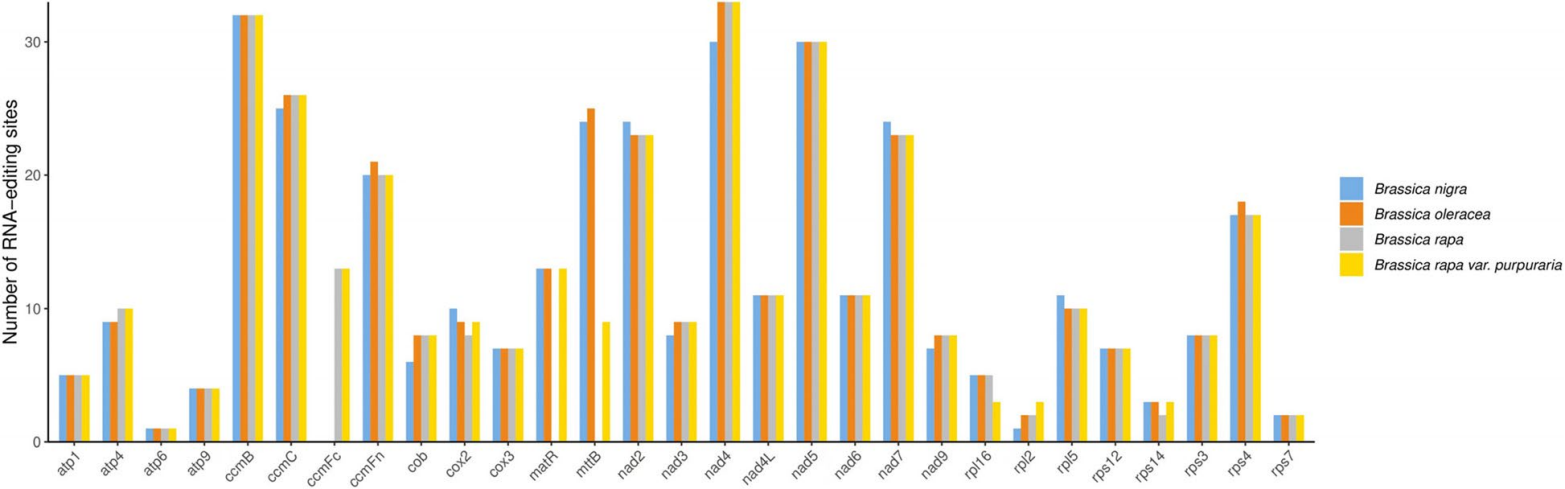
The distribution and comparison of RNA-editing sites in the PCGs of *B. rapa var. Purpuraria* mt genome and three closely related *Brassica* mt genomes

In addition, we compared the RNA editing sites of *B. rapa* (AP017996), *B. nigra*(AP012989), and *B.*oleracea (AP012988) with representatives from *Brassica* species (Fig. 3). The highest edited transcripts were *ccmB* with 32 editing sites in *B. nigra,* and *nad4* with 33 editing sites in *B. rapa, B.oleracea* and *B. rapa* var. *Purpuraria*. From the comparison results of RNA editing sites, we found that *B. rapa* var. *Purpuraria* is highly similar to other three closely related *Brassica* species.

### Repeat sequences analysis

Simple sequence repeats (SSRs), also known as microsatellites, are DNA stretches composing of short unit sequence repeats of 1–6 base pairs in length [43]. In this study, a total of 55 SSRs were detected in the mt genome of *B. rapa* var. *Purpuraria*, containing 20 (36.36%) monomers, 11 (20.00%) dimers, 5(9.09%) trimers, 18 (32.73%) tetramers, and 1 (1.82%) pentamers (Table 4). No hexanucleotide repeats were detected. Among the 55 SSRs, monomer and tetramer were the main type of SSR motifs, accounting for 69.09% of all detected SSRs. In addition, 90.00% of monomers had A/T contents, and 36.36% of dimers were AT/TA (Table S6). The abundant AT content of SSRs supported with the high AT content (54.77%) of the whole mt genome of *B. rapa* var. *Purpuraria*.

**Table 4:** Frequency of identifed SSR motifs in the *B. rapa* var. *Purpuraria* mt genome

Motif Type	Number of repeats														Total	Total Proportion (%)
	3	4	5	6	7	8	9	10	11	12	13	14	15	16		
Monomer	-	-	-	-	-	-	-	14	2	-	1	1	-	2	20	36.36
Dimer	-	-	10	1	-	-	-	-	-	-	-	-	-	-	11	20.00
Trimer	-	4	-	-	-	1	-	-	-	-	-	-	-	-	5	9.09
Tetramer	17	1	-	-	-	-	-	-	-	-	-	-	-	-	18	32.73
Pentamer	1	-	-	-	-	-	-	-	-	-	-	-	-	-	1	1.82
Total	18	5	10	1	0	1	0	14	2	0	1	1	0	2	55	100.00

**Fig. 4:**
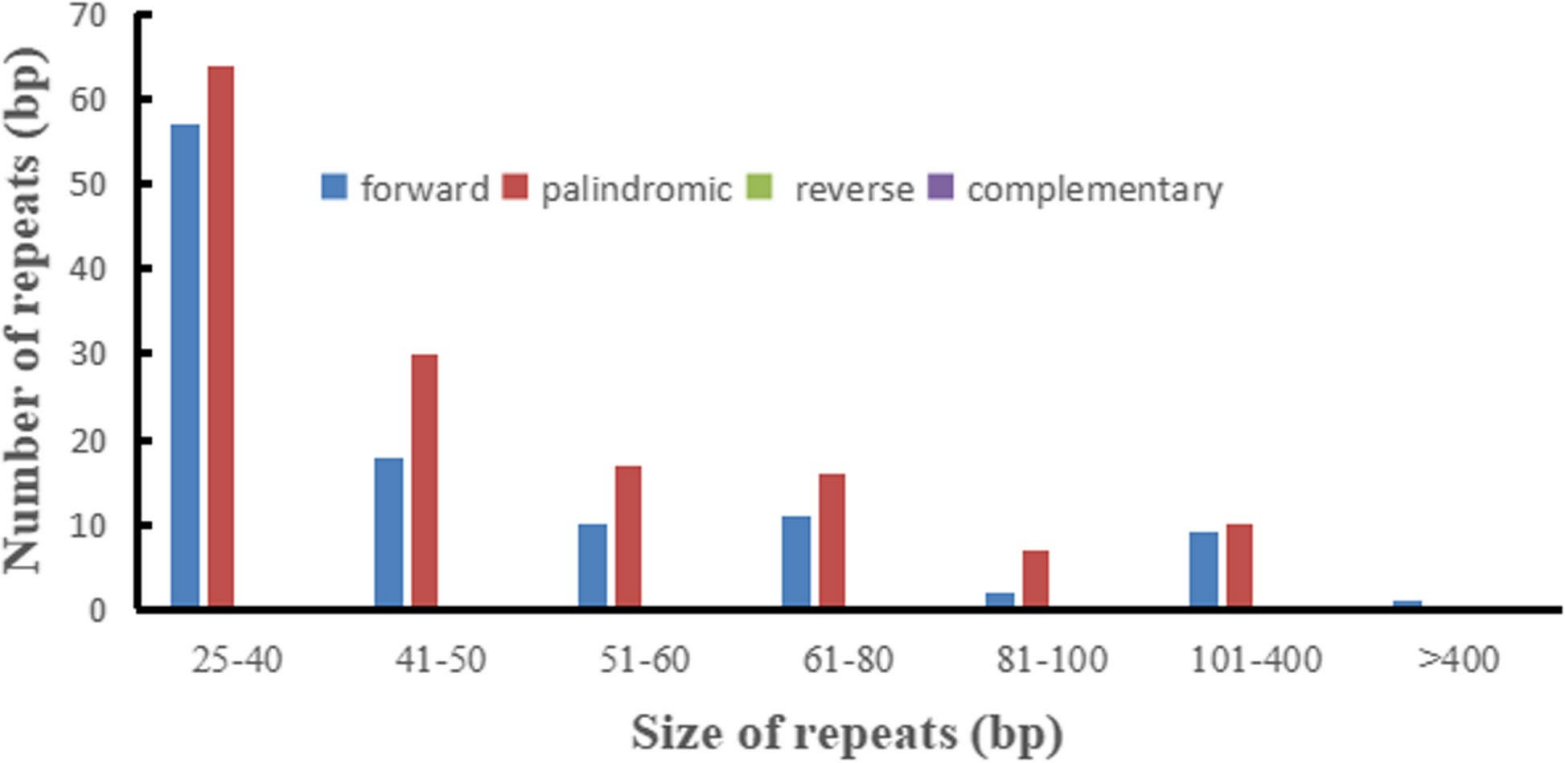
Dispersed repeats analysis in the *B. rapa* var. *Purpuraria* mt genome

Tandem repeats (satellite DNA) are core repeating units about 1—200 bases [44]. As shown in Table 5, 17 tandem repeats with a matching degree over than ≧84% and length varying from 3 to 39 bp were obtained. A total of 252 dispersed repeats (≧28 bp), of which 144 palindromic (57.54%) and 108 forward repeats (42.46%) were observed, and no reverse and complementary repeats were found (Fig. 4). The total length of the dispersed repeats was 16251 bp, which occupied 7.39% of the whole mt genome. Most repeats were 25–40 bp (169 repeats, 67.06%), while only one was over than 1 kb being 2427 bp (Table S7).

**Table 5:** The tandem repeats anaysis of *B. rapa* var. *Purpuraria* mt genome

NO	Chr	Size	Copy	Repeat sequence	Percent Matches	Start	End
1	chr1	3	8.3	CTT	100	4183	4207
2	chr1	29	3.1	AAAGGAGAGGTGCTTTAGCAACTCGACTG	98	28,136	28,225
3	chr1	27	2.7	GCTTTCTTGGTTTGATGAGCTTATACAC	95	49,104	49,177
4	chr1	12	2.1	TGAACTGATAGC	100	61,927	61,951
5	chr1	22	2.2	TCGAGATCTTTGAACCTTTCAG	96	69,117	69,165
6	chr1	36	2.4	AGAAGAAGGTTGACTCGGCAATCTCAATTTCGTATG	94	71,537	71,623
7	chr1	20	1.9	ATGTTAGTGTTCAGTATATC	100	71,739	71,776
8	chr1	12	2.2	CTCGAGGAACGC	100	88,939	88,965
9	chr1	14	2.1	CGGAGGCGGGTAAG	100	116,459	116,488
10	chr1	19	2.1	AGATTTTACAAATGGTCTG	100	118,977	119,015
11	chr1	39	2.8	TATCAATTTCATAAGAGAAGAAAGATCGTTTTTTTAAAT	100	119,157	119,267
12	chr1	17	2.3	ATATATCCATTCTCATA	95	138,230	138,268
13	chr1	18	2.4	TTCCATCAAGAAGGTACC	84	144,510	144,551
14	chr1	15	2	TAGAAAACTGGCATC	100	147,080	147,109
15	chr1	34	2	AAGGAGGAACCCAGCTTATCCCCTCTCAGAGGAG	88	186,199	186,266
16	chr1	15	2.1	TCTTTTCTTGCTCTT	94	191,598	191,629
17	chr1	21	2.7	AACAGATAACAACAGCATATT	100	205,173	205,229

### Ka/Ks and Pi analysis

**Fig. 5:**
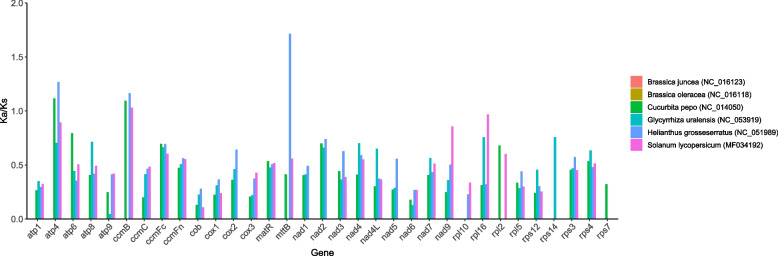
Ka/Ks ratios of 33 PCGs between *B. rapa* var. *Purpuraria* and six species

The Ka/Ks ratio was used to evaluate selective pressures during the evolutionary dynamics of PCGs among similar species. In this work, *B. rapa* var. *Purpuraria* was used as a reference to calculate the Ka/Ks value of 33 PCGs in the *B. rapa* var. *Purpuraria* mt genome (Fig. 5). The Ka/Ks values of most of PCGs were less than one, demonstrating that these genes may undergo negative selections during evolution. However, the Ka/Ks value of the *atp4* and *ccmB* genes between *B. rapa* var. *Purpuraria* and *Cucurbita pepo*, the *ccmB* gene between *B. rapa* var. *Purpuraria* and *Glycyrrhiza uralensis*, the *atp4*, *ccmB*, and *mttB* genes between *B. rapa* var. *Purpuraria* and *Helianthus grosseserratus*, the *ccmB* gene between *B. rapa* var. *Purpuraria* and *Solanum lycopersicum* were higher than one, implying positive selection for these genes during evolution. Our findings further indicated that these mt genes might be highly conserved during the evolution process in higher plants.

**Fig. 6:**
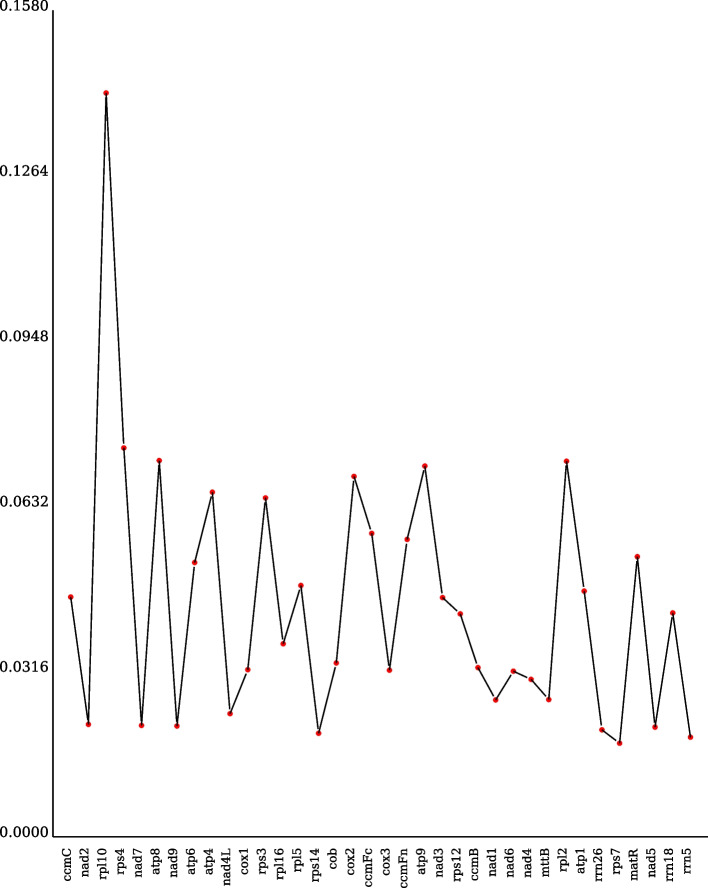
Nucleotide diversity of *B. rapa* var. *Purpuraria* mt genome

The nucleotide diversity (Pi) values of 36 PCGs were accounted and varied from 0.01790 to 0.14222, with an average of 0.04417 (Fig. 6 and Table S8). The Pi value of *gene3.rpl10* region was largest among these regions being 0.1422, and 0.07436 in *gene4.rps4,* 0.07195 in *gene6.atp8,* 0.07182 in *gene29.rpl2* and 0.0709 in *gene21.atp9* were found The lower Pi values revealed that the mt genome sequences of *B. rapa* var. *Purpuraria* were highly conserved.

### Phylogenetic analysis

**Fig. 7:**
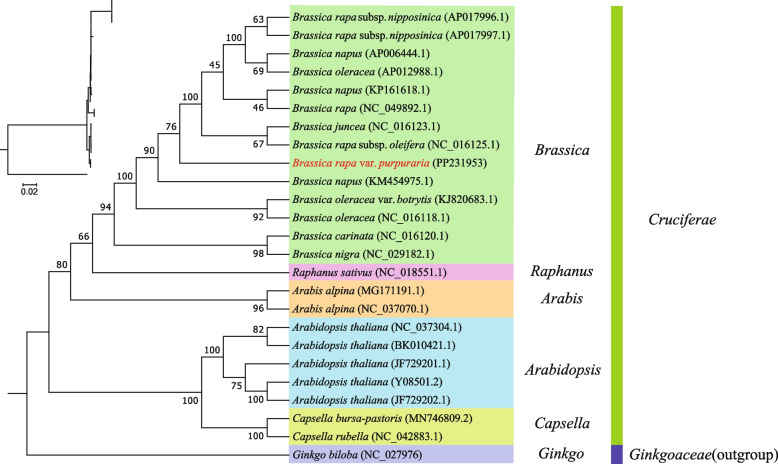
Maximum-likelihood phylogenetic tree based on 31 conserved PCGs among 25 species. *Ginkgo biloba* (NC_027976) used as the out group

To determine the evolutionary status for the mt genome of *B. rapa* var. *Purpuraria*, the phylogenetic analyses was carried out on *B. rapa* var. *Purpuraria* together with other 24 Cruciferae/*Brassica* species (Fig. 7). A phylogenetic tree was built based on an aligned data matrix of 31 conserved PCGs, including *atp1*, *atp4*, *atp6*, *atp8*, *atp9*, *ccmB*, *ccmC*, *ccmFc*, *ccmFn*, *cob*, *cox1*, *cox2*, *cox3*, *mttB*,* nad1*, *nad2*, *nad3*, *nad4*, *nad4L*, *nad5*, *nad6*, *nad7*, *nad9*, *rpl16*, *rpl2*, *rpl5*, *rps12*, *rps14*, *rps3*, *rps4*, and *rps7*, from all tested species. The phylogenetic tree was divided into six groups, being *Brassica*, *Raphanus*, *Arabis*, *Arabidopsis*, *Capsella*, and *Ginkgo*. *B. rapa* var. *Purpuraria* was clustered with the species of genus *Brassica* at first group, and formed sister branches with other related *Brassica* species within the Cruciferae family clade. Furthermore, *B. rapa* var. *Purpuraria* was closely related to *B. rapa subsp. Oleifera* (NC_016125.1) and *B. juncea* (NC_016123.1)*,* indicating that *B. rapa* var. *Purpuraria* belongs to the *Brassica* in the Cruciferae family.

### Analysis of homologous fragments of mitochondria and chloroplasts

The total length of homologous sequences on chloroplasts was 13,153 bp, accounting for 8.57% of the whole cp genome. While the total size of homologous sequences on mitochondria was 8961 bp, accounting for 4.08% of the whole mt genome (Table S9). As shown in Table 6, twenty-two homologous fragments with a total length of 13,325 bp were found. The transfer route of the fragments may occur firstly from the chloroplast to nucleus, and then to the mitochondrion in *B.rapa* var. *Purpuraria*, accounting for 6.06% of the whole mt genome. Eight annotated genes, namely, *trnL-CAA*, *trnN-GTT*, *rrn18*, *trnW-CCA*, *trnD-GTC*, *trnM-CAT*, *ccmC*, and *trnI-AAT*, with high similarity to the mitochondria likely originated from the mt genome. While 17 genes, being *rpoB, ycf2, ycf15, trnL-CAA, rbcL, trnN-GUU, ycf1, psaA, rrn23, rrn16, psaB, trnW-CCA, trnD-GUC, trnP-UGG, trnM-CAU, trnI-CAU,* and *trnI-GAU* with high similarity to the cp genes, might be transferred from cp genome, and only partial sequences of those genes were identified in the mt genome (Table 6). Most of transferred genes were tRNA genes, of which those genes were much more conserved in the mt genome than PCGs during the evolution.

**Table 6:** Fragments transferred from cp to mt in *B.rapa* var. *Purpuraria*

NO	Length	Identity%	Mismatches	Gap	Cp start	Cp end	Mt start	Mt end	Gene (cp)	Gene (mt)
1	2186	98.262	33	1	23,007	25,192	108,053	110,233	*rpoB*^*a*^	
2	1879	97.339	34	6	91,634	93,500	33,492	31,618	*ycf2*^*a*^*,ycf15,trnL-CAA*	*trnL-CAA*
3	1879	97.339	34	6	143,266	145,132	31,618	33,492	*trnL-CAA,ycf15,ycf2*^*a*^	*trnL-CAA*
4	1365	96.41	39	3	53,575	54,934	142,589	141,230	*rbcL*^*a*^	
5	1159	99.655	4	0	108,069	109,227	63,319	62,161	*trnN-GUU,ycf1*^*a*^	*trnN-GTT*
6	1159	99.655	4	0	127,539	128,697	62,161	63,319	*ycf1*^*a*^*,trnN-GUU*	*trnN-GTT*
7	659	96.055	20	3	40,170	40,826	38,184	37,530	*psaA*^*a*^	
8	229	96.943	6	1	103,753	103,980	89,675	89,447	*rrn23*^*a*^	
9	229	96.943	6	1	132,786	133,013	89,447	89,675	*rrn23*^*a*^	
10	878	74.374	170	43	135,617	136,469	210,650	209,803	*rrn16*^*a*^	*rrn18*^*a*^
11	878	74.374	170	43	100,297	101,149	209,803	210,650	*rrn16*^*a*^	*rrn18*^*a*^
12	91	98.901	1	0	38,693	38,783	48,932	49,022	*psaB*^*a*^*,psaA*^*a*^	
13	143	86.713	11	8	65,123	65,264	34,481	34,346	*trnW-CCA*	*trnW-CCA*
14	74	97.297	2	0	29,508	29,581	71,853	71,780	*trnD-GUC*	*trnD-GTC*
15	118	85.593	17	0	65,457	65,574	34,130	34,013	*trnP-UGG*^*a*^	
16	77	92.208	6	0	50,951	51,027	52,691	52,615	*trnM-CAU*	*trnM-CAT*
17	43	100	0	0	100,387	100,429	117,447	117,405	*rrn16*^*a*^	
18	43	100	0	0	136,337	136,379	117,405	117,447	*rrn16*^*a*^	
19	76	85.526	7	3	85,434	85,508	5573	5501	*trnI-CAU*	*ccmC*^*a*^*, trnI-AAT*
20	76	85.526	7	3	151,258	151,332	5501	5573	*trnI-CAU*	*ccmC*^*a*^*, trnI-AAT*
21	42	97.619	1	0	134,348	134,389	17,596	17,555	*trnI-GAU*^*a*^	
22	42	97.619	1	0	102,377	102,418	17,555	17,596	*trnI-GAU*^*a*^	

^*a*^Represents the partial sequence identified in mt genome

## Discussion

Mitochondria is the core of energy source in cells, which exhibited more complex in plant than animals due to its size variations and repetitive sequences [45–47]. In this study, we analyzed the characteristics of mt genome in *B. rapa* var. *Purpuraria*. The total length of the mt genome of *B. rapa* var. *Purpuraria* was similar to that of *B. juncea* [48], being moderate in genome length compared with other reported mt genomes [49]. The GC content in the *B. rapa* var. *Purpuraria* mt genome is 45.23%, which is similar to that of other reported plant mt genomes such as *Camellia sinensis* var. Assamica cv. Duntsa, 45.62% [50]; *Mesona chinensis* Benth, 44.21% [51]; *B. napus*, 45.21% [52], while exhibited greater than the *B. rapa* var. *Purpuraria* cp genome (PP191173, 36.36%) assembled by our research group. Non-coding sequence accounted for 71.7% of the whole *B. rapa* var. *Purpuraria* mt genome, which is similar to most of plant mt genomes [52–54]. In addition, the PCGs occupied 13.22% might be due to the result of increasing sequence repeats during evolution. PCGs usually encoded from initiation codon (ATG) to stop codons (TGA, TAA, and TGA), and the distribution of amino acids compositions was consistent with *Acer yangbiense* [55] and *A. thaliana* [56]. The *nad1* gene using ACG as start codon in consistent with *Salix suchowensis* and *Phaseolus vulgaris* might be induced by RNA editing [46, 47].

Codon usage bias refers that synonymous codons exist in a non-random manner in different species [57]. The analysis of codon usage patterns is helpful to understand the molecular mechanism of biological adaptation and explore the evolutionary relationship among species [58]. Previous studies have shown that codons prefer to use A / U endings in the plant mt genomes [44–47]. In total of 30 high-frequency codons were detected in the *B. rapa* var. *Purpuraria* mt genome, of which 90% codons ended with A or U, which might be the result of natural selection, mutation pressure and genetic drift [59]. In addition, we found that leucine was the most frequently used amino acid, which was consistent with *S. glauca* [44] and *Acer truncatum* Bunge [60].

The number of RNA editing sites varies among different plants, and occurred commonly in gymnosperm and angiosperm mt genomes. We obtained 379 RNA editing sites within all the 33 PCGs in the *B. rapa* var. *Purpuraria* mt genome, which exhibited much less than those in *Diospyros oleifera* (515) [61], *Bupleurum chinense* DC (517) [38], *Macadamia integrifolia* (688), *M. ternifolia* (689) and *M. tetraphylla* (688) [62], and higher than those in *S.glauca* (261)[44] and *Pereskia aculeata* (362) [63]. The selection of RNA editing sites in the *B. rapa* var. *Purpuraria* genome exhibited a high degree of compositional bias. Most of the RNA editing sites were the C-T editing type, being similar as in other plant mt genomes [64–66]. Previous studies showed that about 50% of RNA editing generated at the second bases of the triplet codes [44, 65]. About 68.07% (258) RNA editing sites occurred at the second codon position in the *B. rapa* var. *Purpuraria* mt genome, greater than that at the first codon position (115, 30.34%). In addition, 1.58% (6) RNA editing sites occurred at both of the first and second codon position. The similar phenomenon was observed in the *D. oleifera* mt genome [61]. There is no RNA editing sites predicted at the third codon position in *B. rapa* var. *Purpuraria* mt genome [44, 66].

The repeat sequences, including SSR, tandem and dispersed repeats, were widely distributed in the plant mt genomes [8, 67]. Previous studies have reported that repeat sequences were important for intermolecular recombination, which play a vital role in forming the mt genome [68]. Because of its high variability and recessive inheritance, SSR has been widely used to confirm phylogenetic relationships, genetic diversity studies and species identification [69]. The mt genome of *B. rapa* var. *Purpuraria* contained 55 SSRs, of which 90.00% of monomers being A or T, resulting 54.77% of AT in the *B. rapa* var. *Purpuraria* mt genome. The high AT content in mt genome were also detected in *Scutellaria tsinyunensis* [70]. and *Magnolia biondii* [71]. In addition, 252 dispersed repeats were discovered in this study, which was much greater than *B. oleracea* var. *Italica* in the genus *Brassica* [72].

The ratio of Ka/Ks provides useful information for reconstructing phylogenetic relationships, and contributes to understand the evolutionary dynamics of PCGs among closely related species [73]. Most of mt genes with Ka/Ks ratios < 1 exhibited negative selections, and a few genes with Ka/Ks ratios > 1 showed positive selections during the evolution in plants [44, 61]. The Ka/Ks ratio of *ccmB* gene was greater than 1 in *S. glauca* mt genome exhibited positive selection [44], whereas Five genes, namely, *atp4*, *nad1*, *ccmC*, *mttB*, and *rpl2,* showed positive selections in *Capsicum pubescens Ruiz & Pav* mt genome [74]*.* Two genes, being *mttB* and *rpl5* exhibited positive selections in European-Asian species [75]. However, three genes with Ka/Ks ratios > 1, including *atp4*, *ccmB*, and *mttB,* exhibited positive selections in our study in consistent with previous studies [44, 74, 75], illustrating that these genes might be selected for future researches on the gene selection and phylogenetic of *Brassica* species. Changes in the size and structure of the plant mt genomes have been obviously observed, whereas the functional genes are still conserved [76]. Previous studies indicated that Pi could reveal the variation of nucleic acid sequences in different plants, and the highly variable regions might be selected as potential molecular markers for population genetics [77, 78]. Pi analysis reflected the variation of nucleotide sequences among different species (Fig. 6). Our results revealed that the Pi value of *rpl10* gene was the largest in these regions, illustrating that *rpl10* gene might be used as molecular markers for the mt genome analysis in *B. rapa* var. *purpuraria*. Except for Reclinomonas, plants are the only group of eukaryotes that still remain the *rpl10* gene in their mt genomes [48, 49]. However, the mt *rpl10* gene has been missed in some Brassicaceae species, and replaced by an additional copy of the nuclear gene that normally encodes cp RPL10 protein [79]. Five highly variable regions, being *ccmB*, *ccmFC*, *rps1*, *rps10*, and *rps14,* might be used as molecular markers in *Phaseolus vulgaris* mt genome. In addtional, the overall low Pi values showed that the mt genome sequences of *B. rapa* var. *Purpuraria* were highly conserved.

Phylogenetic analysis of plants have developed to use the complete genome data to construct the relationship among different species [44–48]. Here, the phylogenetic tree was constructed according to the mt genomes of 25 species. *B. rapa* var. *Purpuraria* was well clustered with the species of genus *Brassica* and stayed closely to *B. rapa subsp. Oleifera* and *B. juncea*, suggesting that *B. rapa* var. *Purpuraria* belongs to the *Brassica* species in the Cruciferae family. Shao et al. (2021) found that *B. oleracea* stayed a closely relationship with *B. rapa* subsp. *Campestris* with 100% support rate [80]. Brassicaceae is a superfamily containing over 3800 species, in which *Brassica* is the most important genus as having many important vegetables and oil crops. It has been mentioned that based on cp genome, *B. napus* always clustered with *B. rapa* morphotypes, but did not cluster into a monophyletic group, and were distantly separated by *B. juncea* and *B. oleracea*. The obtained phylogenetic tree revealed a clear phylogenetic relationship among the species. *B. rapa* var. *Purpuraria* as a local vegetable planted around Yangtze River, may also develop some different characteristics in mt genome to regulate the biosynthesis in anthocyanin, and other nutritional compounds. Therefore, assembling and analyzing the mt genomes with those difference genome information will help to better understand their genetic characteristics and selecting their differences for further investigation. DNA sequence transfer between cp and mt genomes has been frequently discovered in plant mt genomes [81]. In higher plants, the total size of transferred DNA ranges from 50 kb (*A. thaliana*) to 1.1 Mb ( *O. sativa* subsp.japonica) depending on the plant species [82]. In total, 13,325 bp of cp DNA has been transferred into the mt genome of *B. rapa* var. *Purpuraria*, accounting for 6.06% of the *B. rapa* var. *Purpuraria* mt genome. In comparison, the proportion in *M. integrifolia*, *Liriodendron tulipifera*, and *Nicotiana tabacum* is 5.4%, 3%, and 2.5%, respectively [38, 71, 83]. About 22 fragments transferred from the cp genome to the mt genome, containing eight annotated genes, with six tRNA genes (*trnL-CAA*, *trnN-GTT*, *trnW-CCA*, *trnD-GTC*, *trnM-CAT*, and *trnI-AAT*), *rrn18*, and *ccmC*. The tRNA genes transferred from cp to mt genomes has been commonly discovered in angiosperms [44, 84, 85]. These results were consistent with the previous study, which showed much more conserved for tRNA genes than the PCGs during the evolution, and tRNA genes played indispensable roles in mt genome [46].

## Conclusions

In this work, we sequenced and successfully assembled the genome with a circular molecule structure in the mt genome of *B. rapa* var. *Purpuraria*. The full length of mt genome for *B. rapa* var. *Purpuraria* was 219,775 bp, containing 59 genes with 33 PCGs, 23 tRNA and 3 rRNA genes. The codon preferences, repeat sequences, RNA editing sites in the *B. rapa* var. *Purpuraria* mt genome have also been analyzed subsequently. Phylogenetic analysis confirmed that *B. rapa* var. *Purpuraria* exhibited a close relationship with *B. oleracea.* Gene conservation between mt and cp genome was also discovered in *B.rapa* var. *Purpuraria* via analyzing gene migration. The Ka/Ks analysis showed that most of the PCGs exhibited negative selections, demonstrating the conservation of these mt genes during the process of evolution. This study provides useful genetic information about the *B. rapa* var. *Purpuraria* mt genome and forms an important theoretical might also help to analyze the genetic variation, systematic evolution, and breeding of *B. rapa* var. *Purpuraria*.

### Supplementary Information

Supplementary Material 1.

Supplementary Material 2.

## Data Availability

The *B. rapa* var. *Purpuraria* mt genome sequence was uploaded in the GenBank database (accession number PP231953).
