# Supplementary material for: Assembly and comparative analysis of the complete mitochondrial genome of Brassica rapa var. Purpuraria
Source: BMC Genomics. 2024 Jun 1;25:546. doi: 10.1186/s12864-024-10457-1 (PMC11143693; doi:10.1186/s12864-024-10457-1)
Supplement: Supplementary file 1 — Supplementary Material 1. [file 12864_2024_10457_MOESM1_ESM.doc]

Table S1.Second-generation sequencing data of the B. rapa var. Purpuraria mt genome.

| ReadSum | BaseSum | Q20(%) | Q30(%) |
| --- | --- | --- | --- |
| 97855158 | 29356547400 | 97.11 | 91.81 |

Table S2. Third-generation sequencing data of the B. rapa var. Purpuraria mt genome.

| Number of reads | Number of bases | Mean read length (bp) | N50 read length (bp) |
| --- | --- | --- | --- |
| 97855158 | 29356547400 | 12674 | 27413 |

Table S3 Genomic features in the *B. rapa* var. *Purpuraria* mt genome

| Feature | A(%) | T(%) | G(%) | C(%) | GC(%) | Size(bp) | Proportion in Genome (%) |
| --- | --- | --- | --- | --- | --- | --- | --- |
| Whole genome | 27.45 | 27.31 | 22.32 | 22.91 | 45.23 | 219775 | 100 |
| PCGs | 26.34 | 31.03 | 21.7 | 20.92 | 42.63 | 29055 | 13.22 |
| cis-spliced introns | 25.61 | 23.31 | 26.86 | 24.22 | 51.08 | 28253 | 12.86 |
| tRNA genes | 22.57 | 25.81 | 28.76 | 22.86 | 51.62 | 1728 | 0.79 |
| rRNA genes | 26.63 | 21.99 | 28.79 | 22.59 | 51.38 | 5144 | 2.34 |
| Non-coding regions | 27.9 | 27.62 | 21.97 | 22.51 | 44.48 | 157583 | 71.7 |

Table S5. RSCU analysis of each amino acid in the B. rapa var. Purpuraria mt genome.

| **Amino Acid** | **Symbol** | **Codon** | **No.** | **RSCU** |
| --- | --- | --- | --- | --- |
| * | Ter | UAA | 16 | 1.4545 |
| * | Ter | UAG | 7 | 0.6364 |
| * | Ter | UGA | 10 | 0.9091 |
| A | Ala | GCA | 154 | 0.9595 |
| A | Ala | GCC | 149 | 0.9283 |
| A | Ala | GCG | 77 | 0.4798 |
| A | Ala | GCU | 262 | 1.6324 |
| C | Cys | UGC | 53 | 0.7737 |
| C | Cys | UGU | 84 | 1.2263 |
| D | Asp | GAC | 98 | 0.6384 |
| D | Asp | GAU | 209 | 1.3616 |
| E | Glu | GAA | 262 | 1.3436 |
| E | Glu | GAG | 128 | 0.6564 |
| F | Phe | UUC | 282 | 0.852 |
| F | Phe | UUU | 380 | 1.148 |
| G | Gly | GGA | 246 | 1.4513 |
| G | Gly | GGC | 87 | 0.5133 |
| G | Gly | GGG | 121 | 0.7139 |
| G | Gly | GGU | 224 | 1.3215 |
| H | His | CAC | 50 | 0.4219 |
| H | His | CAU | 187 | 1.5781 |
| I | Ile | AUA | 207 | 0.8381 |
| I | Ile | AUC | 206 | 0.834 |
| I | Ile | AUU | 328 | 1.3279 |
| K | Lys | AAA | 253 | 1.1962 |
| K | Lys | AAG | 170 | 0.8038 |
| L | Leu | CUA | 157 | 0.8946 |
| L | Leu | CUC | 110 | 0.6268 |
| L | Leu | CUG | 101 | 0.5755 |
| L | Leu | CUU | 218 | 1.2422 |
| L | Leu | UUA | 265 | 1.51 |
| L | Leu | UUG | 202 | 1.151 |
| M | Met | AUG | 270 | 3 |
| N | Asn | AAC | 93 | 0.6059 |
| N | Asn | AAU | 214 | 1.3941 |
| P | Pro | CCA | 145 | 1.109 |
| P | Pro | CCC | 96 | 0.7342 |
| P | Pro | CCG | 83 | 0.6348 |
| P | Pro | CCU | 199 | 1.522 |
| Q | Gln | CAA | 216 | 1.554 |
| Q | Gln | CAG | 62 | 0.446 |
| R | Arg | AGA | 157 | 1.5072 |
| R | Arg | AGG | 85 | 0.816 |
| R | Arg | CGA | 137 | 1.3152 |
| R | Arg | CGC | 54 | 0.5184 |
| R | Arg | CGG | 65 | 0.624 |
| R | Arg | CGU | 127 | 1.2192 |
| S | Ser | AGC | 102 | 0.715 |
| S | Ser | AGU | 157 | 1.1005 |
| S | Ser | UCA | 147 | 1.0304 |
| S | Ser | UCC | 138 | 0.9673 |
| S | Ser | UCG | 117 | 0.8201 |
| S | Ser | UCU | 195 | 1.3668 |
| T | Thr | ACA | 110 | 0.9148 |
| T | Thr | ACC | 123 | 1.0229 |
| T | Thr | ACG | 73 | 0.6071 |
| T | Thr | ACU | 175 | 1.4553 |
| V | Val | GUA | 186 | 1.2441 |
| V | Val | GUC | 113 | 0.7559 |
| V | Val | GUG | 127 | 0.8495 |
| V | Val | GUU | 172 | 1.1505 |
| W | Trp | UGG | 147 | 1 |
| Y | Tyr | UAC | 73 | 0.4916 |
| Y | Tyr | UAU | 224 | 1.5084 |

Table S6. The identified SSR motifs in the B. rapa var. Purpuraria mt genome.

| Motif Type | Repeats | Number |
| --- | --- | --- |
| Monomer | A | 12 |
|  | C | 2 |
|  | T | 6 |
| Dimer | AG | 2 |
|  | AT | 2 |
|  | CT | 1 |
|  | TA | 2 |
|  | TC | 4 |
| Trimer | CTT | 1 |
|  | GTT | 1 |
|  | TTC | 3 |
| Tetramer | AAAC | 2 |
|  | AAAT | 1 |
|  | AAGA | 3 |
|  | AATG | 2 |
|  | AGTG | 1 |
|  | CATT | 1 |
|  | GCCG | 1 |
|  | GTTC | 1 |
|  | TCAT | 1 |
|  | TTCA | 1 |
|  | TTCT | 1 |
|  | TTTA | 2 |
|  | TTTG | 1 |
| Pentamer | CTAGT | 1 |

Table S7. Dispersed repeats analysis in the B. rapa var. Purpuraria mt genome.

| #Chr1 | Chr2 | type | length | similarity | start1 | end1 | start2 | end2 | evalue |
| --- | --- | --- | --- | --- | --- | --- | --- | --- | --- |
| chr1 | chr1 | F | 2427 | 100 | 82649 | 85075 | 1 | 2427 | 0 |
| chr1 | chr1 | P | 308 | 99.675 | 175350 | 175657 | 95989 | 96296 | 6.28E-160 |
| chr1 | chr1 | F | 312 | 99.038 | 199773 | 200083 | 112553 | 112864 | 2.92E-158 |
| chr1 | chr1 | F | 268 | 96.269 | 140841 | 141108 | 58916 | 59183 | 1.10E-122 |
| chr1 | chr1 | P | 232 | 99.569 | 185864 | 186095 | 119231 | 119462 | 1.11E-117 |
| chr1 | chr1 | P | 284 | 91.197 | 112766 | 113046 | 27732 | 28009 | 8.78E-104 |
| chr1 | chr1 | P | 152 | 98.684 | 120807 | 120957 | 37177 | 37328 | 5.52E-71 |
| chr1 | chr1 | P | 147 | 99.32 | 214695 | 214841 | 203231 | 203376 | 7.13E-70 |
| chr1 | chr1 | F | 140 | 98.571 | 60815 | 60954 | 28035 | 28174 | 7.18E-65 |
| chr1 | chr1 | F | 132 | 100 | 110636 | 110767 | 25879 | 26010 | 9.29E-64 |
| chr1 | chr1 | P | 254 | 85.433 | 169939 | 170191 | 26849 | 27077 | 1.20E-62 |
| chr1 | chr1 | F | 168 | 92.857 | 165792 | 165957 | 25907 | 26071 | 4.32E-62 |
| chr1 | chr1 | P | 99 | 95.96 | 199985 | 200083 | 27911 | 28009 | 9.63E-39 |
| chr1 | chr1 | P | 110 | 91.818 | 85175 | 85284 | 42372 | 42481 | 1.61E-36 |
| chr1 | chr1 | F | 106 | 91.509 | 209747 | 209852 | 119414 | 119519 | 2.70E-34 |
| chr1 | chr1 | P | 78 | 100 | 163891 | 163968 | 51696 | 51773 | 9.70E-34 |
| chr1 | chr1 | P | 78 | 100 | 165949 | 166026 | 126072 | 126149 | 9.70E-34 |
| chr1 | chr1 | P | 154 | 84.416 | 138305 | 138443 | 6663 | 6816 | 1.62E-31 |
| chr1 | chr1 | F | 79 | 97.468 | 199671 | 199749 | 103557 | 103635 | 5.84E-31 |
| chr1 | chr1 | F | 76 | 98.684 | 119192 | 119267 | 119153 | 119228 | 5.84E-31 |
| chr1 | chr1 | P | 81 | 96.296 | 211312 | 211392 | 27081 | 27161 | 2.10E-30 |
| chr1 | chr1 | F | 91 | 93.407 | 167960 | 168050 | 36902 | 36991 | 2.10E-30 |
| chr1 | chr1 | P | 79 | 97.468 | 219086 | 219164 | 114511 | 114588 | 2.10E-30 |
| chr1 | chr1 | F | 107 | 88.785 | 165792 | 165896 | 110664 | 110767 | 3.51E-28 |
| chr1 | chr1 | P | 62 | 100 | 186059 | 186120 | 119128 | 119189 | 7.60E-25 |
| chr1 | chr1 | P | 71 | 94.366 | 69039 | 69109 | 28198 | 28265 | 4.58E-22 |
| chr1 | chr1 | F | 65 | 95.385 | 175657 | 175721 | 51621 | 51685 | 1.65E-21 |
| chr1 | chr1 | F | 62 | 96.774 | 200601 | 200662 | 74081 | 74142 | 1.65E-21 |
| chr1 | chr1 | P | 73 | 93.151 | 215740 | 215807 | 16921 | 16993 | 5.92E-21 |
| chr1 | chr1 | F | 56 | 98.214 | 147778 | 147833 | 13707 | 13762 | 7.66E-20 |
| chr1 | chr1 | P | 59 | 96.61 | 149299 | 149357 | 41313 | 41371 | 7.66E-20 |
| chr1 | chr1 | P | 65 | 93.846 | 41313 | 41377 | 149293 | 149357 | 7.66E-20 |
| chr1 | chr1 | P | 59 | 96.61 | 77300 | 77358 | 12120 | 12177 | 2.75E-19 |
| chr1 | chr1 | P | 117 | 82.906 | 218623 | 218737 | 43633 | 43741 | 2.75E-19 |
| chr1 | chr1 | P | 54 | 98.148 | 199359 | 199412 | 187465 | 187518 | 9.91E-19 |
| chr1 | chr1 | P | 57 | 96.491 | 187465 | 187521 | 199356 | 199412 | 9.91E-19 |
| chr1 | chr1 | P | 53 | 98.113 | 51724 | 51776 | 29074 | 29126 | 3.56E-18 |
| chr1 | chr1 | F | 50 | 100 | 163891 | 163940 | 29077 | 29126 | 3.56E-18 |
| chr1 | chr1 | P | 50 | 100 | 29077 | 29126 | 51724 | 51773 | 3.56E-18 |
| chr1 | chr1 | P | 49 | 100 | 101871 | 101919 | 10307 | 10355 | 1.28E-17 |
| chr1 | chr1 | P | 59 | 94.915 | 79059 | 79117 | 28186 | 28243 | 1.28E-17 |
| chr1 | chr1 | P | 65 | 92.308 | 28186 | 28249 | 79053 | 79117 | 1.28E-17 |
| chr1 | chr1 | P | 58 | 94.828 | 60936 | 60992 | 23558 | 23614 | 4.61E-17 |
| chr1 | chr1 | F | 51 | 98.039 | 145779 | 145829 | 119671 | 119721 | 4.61E-17 |
| chr1 | chr1 | P | 150 | 78 | 218621 | 218766 | 131858 | 132006 | 4.61E-17 |
| chr1 | chr1 | F | 79 | 87.342 | 71660 | 71738 | 33571 | 33646 | 1.66E-16 |
| chr1 | chr1 | P | 52 | 96.154 | 4083 | 4134 | 1884 | 1935 | 5.96E-16 |
| chr1 | chr1 | P | 58 | 93.103 | 84532 | 84589 | 4077 | 4134 | 5.96E-16 |
| chr1 | chr1 | P | 58 | 93.103 | 1884 | 1941 | 4077 | 4134 | 5.96E-16 |
| chr1 | chr1 | P | 52 | 96.154 | 65599 | 65650 | 21799 | 21850 | 5.96E-16 |
| chr1 | chr1 | P | 46 | 100 | 199111 | 199156 | 74450 | 74495 | 5.96E-16 |
| chr1 | chr1 | P | 52 | 96.154 | 4083 | 4134 | 84532 | 84583 | 5.96E-16 |
| chr1 | chr1 | P | 85 | 84.706 | 141116 | 141200 | 112025 | 112107 | 2.14E-15 |
| chr1 | chr1 | P | 82 | 85.366 | 112028 | 112107 | 141116 | 141197 | 2.14E-15 |
| chr1 | chr1 | F | 45 | 100 | 166887 | 166931 | 160014 | 160058 | 2.14E-15 |
| chr1 | chr1 | F | 48 | 97.917 | 71660 | 71707 | 65573 | 65619 | 7.71E-15 |
| chr1 | chr1 | P | 71 | 87.324 | 69039 | 69109 | 60920 | 60988 | 2.77E-14 |
| chr1 | chr1 | F | 65 | 89.231 | 79061 | 79125 | 69066 | 69128 | 2.77E-14 |
| chr1 | chr1 | P | 49 | 95.918 | 209747 | 209795 | 185864 | 185912 | 2.77E-14 |
| chr1 | chr1 | P | 45 | 97.778 | 51355 | 51399 | 4046 | 4090 | 9.98E-14 |
| chr1 | chr1 | F | 52 | 94.231 | 71573 | 71623 | 71537 | 71588 | 9.98E-14 |
| chr1 | chr1 | F | 72 | 87.5 | 185669 | 185735 | 141106 | 141177 | 9.98E-14 |
| chr1 | chr1 | F | 51 | 94.118 | 65568 | 65617 | 33566 | 33615 | 3.59E-13 |
| chr1 | chr1 | P | 41 | 100 | 170361 | 170401 | 154872 | 154912 | 3.59E-13 |
| chr1 | chr1 | P | 41 | 100 | 212101 | 212141 | 170360 | 170400 | 3.59E-13 |
| chr1 | chr1 | F | 45 | 97.778 | 215037 | 215080 | 209251 | 209295 | 3.59E-13 |
| chr1 | chr1 | F | 40 | 100 | 44660 | 44699 | 16929 | 16968 | 1.29E-12 |
| chr1 | chr1 | F | 50 | 94 | 60905 | 60954 | 28156 | 28203 | 1.29E-12 |
| chr1 | chr1 | P | 40 | 100 | 215760 | 215799 | 44660 | 44699 | 1.29E-12 |
| chr1 | chr1 | F | 50 | 94 | 49129 | 49177 | 49101 | 49150 | 1.29E-12 |
| chr1 | chr1 | F | 40 | 100 | 212101 | 212140 | 154873 | 154912 | 1.29E-12 |
| chr1 | chr1 | F | 39 | 100 | 159776 | 159814 | 29079 | 29117 | 4.64E-12 |
| chr1 | chr1 | P | 39 | 100 | 212105 | 212143 | 51693 | 51731 | 4.64E-12 |
| chr1 | chr1 | P | 42 | 97.619 | 159776 | 159817 | 51730 | 51771 | 4.64E-12 |
| chr1 | chr1 | P | 86 | 82.558 | 113770 | 113854 | 57125 | 57207 | 4.64E-12 |
| chr1 | chr1 | F | 42 | 97.619 | 212101 | 212142 | 101454 | 101495 | 4.64E-12 |
| chr1 | chr1 | F | 46 | 95.652 | 163886 | 163931 | 159770 | 159814 | 4.64E-12 |
| chr1 | chr1 | P | 42 | 97.619 | 51733 | 51774 | 159773 | 159814 | 4.64E-12 |
| chr1 | chr1 | P | 42 | 97.619 | 60920 | 60960 | 305 | 346 | 1.67E-11 |
| chr1 | chr1 | P | 67 | 86.567 | 74080 | 74143 | 22209 | 22275 | 1.67E-11 |
| chr1 | chr1 | P | 45 | 95.556 | 154873 | 154916 | 29882 | 29926 | 1.67E-11 |
| chr1 | chr1 | P | 41 | 97.561 | 212101 | 212141 | 29886 | 29926 | 1.67E-11 |
| chr1 | chr1 | F | 41 | 97.561 | 170360 | 170400 | 29886 | 29926 | 1.67E-11 |
| chr1 | chr1 | P | 47 | 93.617 | 85094 | 85140 | 42445 | 42491 | 1.67E-11 |
| chr1 | chr1 | F | 78 | 83.333 | 131888 | 131964 | 43633 | 43709 | 1.67E-11 |
| chr1 | chr1 | P | 42 | 97.619 | 82953 | 82994 | 60920 | 60960 | 1.67E-11 |
| chr1 | chr1 | P | 67 | 86.567 | 187535 | 187598 | 60923 | 60988 | 1.67E-11 |
| chr1 | chr1 | P | 38 | 100 | 42454 | 42491 | 85094 | 85131 | 1.67E-11 |
| chr1 | chr1 | F | 41 | 97.561 | 164664 | 164704 | 100377 | 100417 | 1.67E-11 |
| chr1 | chr1 | P | 41 | 97.561 | 170360 | 170400 | 101454 | 101494 | 1.67E-11 |
| chr1 | chr1 | P | 66 | 87.879 | 163635 | 163692 | 103108 | 103173 | 1.67E-11 |
| chr1 | chr1 | P | 56 | 89.286 | 205095 | 205150 | 179320 | 179375 | 1.67E-11 |
| chr1 | chr1 | P | 53 | 90.566 | 179323 | 179375 | 205095 | 205147 | 1.67E-11 |
| chr1 | chr1 | P | 40 | 97.5 | 79078 | 79117 | 28157 | 28196 | 6.00E-11 |
| chr1 | chr1 | F | 53 | 90.566 | 88108 | 88158 | 29079 | 29131 | 6.00E-11 |
| chr1 | chr1 | P | 43 | 95.349 | 154877 | 154919 | 51689 | 51731 | 6.00E-11 |
| chr1 | chr1 | F | 37 | 100 | 170360 | 170396 | 51695 | 51731 | 6.00E-11 |
| chr1 | chr1 | P | 68 | 85.294 | 165543 | 165610 | 77304 | 77370 | 6.00E-11 |
| chr1 | chr1 | F | 47 | 93.617 | 154867 | 154912 | 101447 | 101493 | 6.00E-11 |
| chr1 | chr1 | P | 40 | 97.5 | 51692 | 51731 | 154877 | 154916 | 6.00E-11 |
| chr1 | chr1 | F | 37 | 100 | 163933 | 163969 | 154877 | 154913 | 6.00E-11 |
| chr1 | chr1 | F | 47 | 93.617 | 170361 | 170406 | 160367 | 160412 | 6.00E-11 |
| chr1 | chr1 | P | 43 | 95.349 | 28260 | 28300 | 304 | 346 | 2.16E-10 |
| chr1 | chr1 | P | 43 | 95.349 | 82952 | 82994 | 28260 | 28300 | 2.16E-10 |
| chr1 | chr1 | P | 36 | 100 | 121081 | 121116 | 48876 | 48911 | 2.16E-10 |
| chr1 | chr1 | P | 39 | 97.436 | 48876 | 48914 | 121078 | 121116 | 2.16E-10 |
| chr1 | chr1 | F | 36 | 100 | 160308 | 160343 | 159071 | 159106 | 2.16E-10 |
| chr1 | chr1 | P | 47 | 93.617 | 212097 | 212140 | 160367 | 160413 | 2.16E-10 |
| chr1 | chr1 | P | 36 | 100 | 170361 | 170396 | 163933 | 163968 | 2.16E-10 |
| chr1 | chr1 | F | 36 | 100 | 212105 | 212140 | 163933 | 163968 | 2.16E-10 |
| chr1 | chr1 | P | 35 | 100 | 28169 | 28203 | 312 | 346 | 7.77E-10 |
| chr1 | chr1 | P | 35 | 100 | 28140 | 28174 | 312 | 346 | 7.77E-10 |
| chr1 | chr1 | P | 35 | 100 | 65599 | 65633 | 6201 | 6235 | 7.77E-10 |
| chr1 | chr1 | F | 35 | 100 | 28260 | 28294 | 28140 | 28174 | 7.77E-10 |
| chr1 | chr1 | P | 35 | 100 | 82960 | 82994 | 28140 | 28174 | 7.77E-10 |
| chr1 | chr1 | F | 35 | 100 | 28260 | 28294 | 28169 | 28203 | 7.77E-10 |
| chr1 | chr1 | P | 35 | 100 | 82960 | 82994 | 28169 | 28203 | 7.77E-10 |
| chr1 | chr1 | F | 93 | 80.645 | 60905 | 60995 | 28185 | 28272 | 7.77E-10 |
| chr1 | chr1 | F | 35 | 100 | 60920 | 60954 | 28260 | 28294 | 7.77E-10 |
| chr1 | chr1 | P | 41 | 95.122 | 101454 | 101494 | 29886 | 29926 | 7.77E-10 |
| chr1 | chr1 | P | 38 | 97.368 | 101458 | 101495 | 51694 | 51731 | 7.77E-10 |
| chr1 | chr1 | P | 35 | 100 | 88119 | 88153 | 51724 | 51758 | 7.77E-10 |
| chr1 | chr1 | P | 42 | 95.238 | 6201 | 6240 | 65592 | 65633 | 7.77E-10 |
| chr1 | chr1 | F | 35 | 100 | 95345 | 95379 | 77374 | 77408 | 7.77E-10 |
| chr1 | chr1 | F | 48 | 91.667 | 163893 | 163940 | 88108 | 88153 | 7.77E-10 |
| chr1 | chr1 | P | 48 | 91.667 | 51724 | 51771 | 88108 | 88153 | 7.77E-10 |
| chr1 | chr1 | P | 42 | 95.238 | 160367 | 160408 | 154873 | 154912 | 7.77E-10 |
| chr1 | chr1 | F | 35 | 100 | 187376 | 187410 | 186336 | 186370 | 7.77E-10 |
| chr1 | chr1 | F | 53 | 88.679 | 175876 | 175927 | 2262 | 2314 | 2.79E-09 |
| chr1 | chr1 | F | 41 | 95.122 | 51692 | 51731 | 29882 | 29922 | 2.79E-09 |
| chr1 | chr1 | F | 34 | 100 | 206048 | 206081 | 37438 | 37471 | 2.79E-09 |
| chr1 | chr1 | F | 34 | 100 | 160367 | 160400 | 51696 | 51729 | 2.79E-09 |
| chr1 | chr1 | F | 44 | 93.182 | 79046 | 79088 | 68978 | 69020 | 2.79E-09 |
| chr1 | chr1 | F | 53 | 88.679 | 175876 | 175927 | 84910 | 84962 | 2.79E-09 |
| chr1 | chr1 | F | 37 | 97.297 | 85175 | 85211 | 85104 | 85140 | 2.79E-09 |
| chr1 | chr1 | F | 37 | 97.297 | 119231 | 119267 | 119153 | 119189 | 2.79E-09 |
| chr1 | chr1 | P | 37 | 97.297 | 186059 | 186095 | 119192 | 119228 | 2.79E-09 |
| chr1 | chr1 | P | 44 | 93.182 | 163935 | 163976 | 160357 | 160400 | 2.79E-09 |
| chr1 | chr1 | P | 34 | 100 | 160367 | 160400 | 163935 | 163968 | 2.79E-09 |
| chr1 | chr1 | F | 77 | 81.818 | 170754 | 170829 | 167669 | 167745 | 2.79E-09 |
| chr1 | chr1 | P | 33 | 100 | 195542 | 195574 | 6205 | 6237 | 1.00E-08 |
| chr1 | chr1 | F | 37 | 97.297 | 187857 | 187892 | 6924 | 6960 | 1.00E-08 |
| chr1 | chr1 | F | 106 | 78.302 | 28185 | 28287 | 28125 | 28225 | 1.00E-08 |
| chr1 | chr1 | F | 43 | 93.023 | 160367 | 160409 | 29887 | 29927 | 1.00E-08 |
| chr1 | chr1 | P | 36 | 97.222 | 163933 | 163968 | 29887 | 29922 | 1.00E-08 |
| chr1 | chr1 | F | 36 | 97.222 | 163933 | 163968 | 101458 | 101493 | 1.00E-08 |
| chr1 | chr1 | F | 36 | 97.222 | 211707 | 211742 | 137708 | 137743 | 1.00E-08 |
| chr1 | chr1 | P | 39 | 94.872 | 54374 | 54412 | 310 | 347 | 3.61E-08 |
| chr1 | chr1 | F | 35 | 97.143 | 21816 | 21850 | 6201 | 6235 | 3.61E-08 |
| chr1 | chr1 | F | 32 | 100 | 111199 | 111230 | 16743 | 16774 | 3.61E-08 |
| chr1 | chr1 | P | 38 | 94.737 | 199346 | 199383 | 23581 | 23618 | 3.61E-08 |
| chr1 | chr1 | P | 41 | 92.683 | 43400 | 43440 | 28166 | 28206 | 3.61E-08 |
| chr1 | chr1 | P | 39 | 94.872 | 187561 | 187598 | 28172 | 28209 | 3.61E-08 |
| chr1 | chr1 | F | 114 | 76.316 | 104759 | 104871 | 37394 | 37507 | 3.61E-08 |
| chr1 | chr1 | P | 32 | 100 | 69080 | 69111 | 54373 | 54404 | 3.61E-08 |
| chr1 | chr1 | P | 39 | 94.872 | 82958 | 82995 | 54374 | 54412 | 3.61E-08 |
| chr1 | chr1 | F | 77 | 81.818 | 187526 | 187598 | 69031 | 69106 | 3.61E-08 |
| chr1 | chr1 | F | 35 | 97.143 | 217760 | 217794 | 78816 | 78850 | 3.61E-08 |
| chr1 | chr1 | F | 32 | 100 | 209577 | 209608 | 100331 | 100362 | 3.61E-08 |
| chr1 | chr1 | P | 42 | 92.857 | 160367 | 160408 | 101454 | 101493 | 3.61E-08 |
| chr1 | chr1 | F | 32 | 100 | 147701 | 147732 | 106406 | 106437 | 3.61E-08 |
| chr1 | chr1 | F | 39 | 94.872 | 202110 | 202148 | 191671 | 191708 | 3.61E-08 |
| chr1 | chr1 | P | 38 | 94.737 | 23584 | 23621 | 199343 | 199380 | 3.61E-08 |
| chr1 | chr1 | F | 41 | 92.683 | 209453 | 209493 | 209339 | 209379 | 3.61E-08 |
| chr1 | chr1 | F | 40 | 92.5 | 219736 | 219775 | 1766 | 1805 | 1.30E-07 |
| chr1 | chr1 | P | 31 | 100 | 29638 | 29668 | 23595 | 23625 | 1.30E-07 |
| chr1 | chr1 | P | 31 | 100 | 79078 | 79108 | 28137 | 28167 | 1.30E-07 |
| chr1 | chr1 | F | 34 | 97.059 | 196513 | 196546 | 28139 | 28172 | 1.30E-07 |
| chr1 | chr1 | F | 34 | 97.059 | 196513 | 196546 | 28168 | 28201 | 1.30E-07 |
| chr1 | chr1 | P | 34 | 97.059 | 80992 | 81025 | 40637 | 40670 | 1.30E-07 |
| chr1 | chr1 | F | 38 | 94.737 | 60920 | 60956 | 54375 | 54412 | 1.30E-07 |
| chr1 | chr1 | P | 31 | 100 | 79078 | 79108 | 60917 | 60947 | 1.30E-07 |
| chr1 | chr1 | F | 34 | 97.059 | 196513 | 196546 | 60919 | 60952 | 1.30E-07 |
| chr1 | chr1 | P | 57 | 85.965 | 132469 | 132522 | 64245 | 64301 | 1.30E-07 |
| chr1 | chr1 | F | 31 | 100 | 195544 | 195574 | 65599 | 65629 | 1.30E-07 |
| chr1 | chr1 | P | 31 | 100 | 40640 | 40670 | 80992 | 81022 | 1.30E-07 |
| chr1 | chr1 | F | 40 | 92.5 | 219736 | 219775 | 84414 | 84453 | 1.30E-07 |
| chr1 | chr1 | P | 31 | 100 | 186071 | 186101 | 111917 | 111947 | 1.30E-07 |
| chr1 | chr1 | F | 31 | 100 | 119147 | 119177 | 111917 | 111947 | 1.30E-07 |
| chr1 | chr1 | F | 38 | 94.737 | 195539 | 195576 | 147639 | 147675 | 1.30E-07 |
| chr1 | chr1 | F | 33 | 96.97 | 23229 | 23261 | 312 | 344 | 4.67E-07 |
| chr1 | chr1 | P | 33 | 96.97 | 196514 | 196546 | 314 | 346 | 4.67E-07 |
| chr1 | chr1 | P | 81 | 80.247 | 167812 | 167886 | 2319 | 2399 | 4.67E-07 |
| chr1 | chr1 | F | 36 | 94.444 | 165575 | 165610 | 12139 | 12174 | 4.67E-07 |
| chr1 | chr1 | P | 65 | 83.077 | 200601 | 200662 | 22210 | 22274 | 4.67E-07 |
| chr1 | chr1 | F | 33 | 96.97 | 82960 | 82992 | 23229 | 23261 | 4.67E-07 |
| chr1 | chr1 | P | 33 | 96.97 | 60922 | 60954 | 23229 | 23261 | 4.67E-07 |
| chr1 | chr1 | P | 33 | 96.97 | 28262 | 28294 | 23229 | 23261 | 4.67E-07 |
| chr1 | chr1 | P | 33 | 96.97 | 28171 | 28203 | 23229 | 23261 | 4.67E-07 |
| chr1 | chr1 | P | 33 | 96.97 | 28142 | 28174 | 23229 | 23261 | 4.67E-07 |
| chr1 | chr1 | P | 39 | 92.308 | 43402 | 43440 | 28137 | 28175 | 4.67E-07 |
| chr1 | chr1 | P | 34 | 97.059 | 187565 | 187598 | 28143 | 28175 | 4.67E-07 |
| chr1 | chr1 | F | 33 | 96.97 | 196514 | 196546 | 28260 | 28292 | 4.67E-07 |
| chr1 | chr1 | P | 30 | 100 | 39447 | 39476 | 36216 | 36245 | 4.67E-07 |
| chr1 | chr1 | P | 30 | 100 | 79076 | 79105 | 54375 | 54404 | 4.67E-07 |
| chr1 | chr1 | P | 33 | 96.97 | 187566 | 187598 | 54378 | 54410 | 4.67E-07 |
| chr1 | chr1 | P | 33 | 96.97 | 196514 | 196546 | 82962 | 82994 | 4.67E-07 |
| chr1 | chr1 | P | 81 | 80.247 | 167812 | 167886 | 84967 | 85047 | 4.67E-07 |
| chr1 | chr1 | P | 51 | 86.275 | 167801 | 167851 | 120210 | 120260 | 4.67E-07 |
| chr1 | chr1 | P | 34 | 97.059 | 187843 | 187875 | 147805 | 147838 | 4.67E-07 |
| chr1 | chr1 | P | 75 | 81.333 | 84976 | 85050 | 167809 | 167877 | 4.67E-07 |
| chr1 | chr1 | P | 75 | 81.333 | 2328 | 2402 | 167809 | 167877 | 4.67E-07 |
| chr1 | chr1 | F | 74 | 81.081 | 209109 | 209180 | 205977 | 206048 | 4.67E-07 |
| chr1 | chr1 | F | 33 | 96.97 | 187566 | 187598 | 312 | 343 | 1.68E-06 |
| chr1 | chr1 | F | 29 | 100 | 69082 | 69110 | 319 | 347 | 1.68E-06 |
| chr1 | chr1 | F | 35 | 94.286 | 151550 | 151584 | 975 | 1009 | 1.68E-06 |
| chr1 | chr1 | F | 58 | 84.483 | 42093 | 42147 | 2161 | 2217 | 1.68E-06 |
| chr1 | chr1 | P | 32 | 96.875 | 147642 | 147673 | 6205 | 6236 | 1.68E-06 |
| chr1 | chr1 | P | 29 | 100 | 187847 | 187875 | 13734 | 13762 | 1.68E-06 |
| chr1 | chr1 | F | 29 | 100 | 129058 | 129086 | 15100 | 15128 | 1.68E-06 |
| chr1 | chr1 | P | 32 | 96.875 | 50606 | 50637 | 23595 | 23626 | 1.68E-06 |
| chr1 | chr1 | F | 36 | 94.444 | 54375 | 54410 | 28140 | 28174 | 1.68E-06 |
| chr1 | chr1 | F | 36 | 94.444 | 54375 | 54410 | 28169 | 28203 | 1.68E-06 |
| chr1 | chr1 | F | 36 | 94.444 | 54375 | 54410 | 28260 | 28294 | 1.68E-06 |
| chr1 | chr1 | P | 33 | 96.97 | 187566 | 187598 | 28263 | 28294 | 1.68E-06 |
| chr1 | chr1 | F | 32 | 96.875 | 50607 | 50638 | 29638 | 29669 | 1.68E-06 |
| chr1 | chr1 | F | 29 | 100 | 176341 | 176369 | 35925 | 35953 | 1.68E-06 |
| chr1 | chr1 | F | 58 | 84.483 | 84809 | 84865 | 42093 | 42147 | 1.68E-06 |
| chr1 | chr1 | P | 38 | 92.105 | 60917 | 60954 | 43403 | 43440 | 1.68E-06 |
| chr1 | chr1 | F | 36 | 94.444 | 196507 | 196541 | 54367 | 54402 | 1.68E-06 |
| chr1 | chr1 | F | 29 | 100 | 82967 | 82995 | 69082 | 69110 | 1.68E-06 |
| chr1 | chr1 | P | 29 | 100 | 91924 | 91952 | 69647 | 69675 | 1.68E-06 |
| chr1 | chr1 | F | 57 | 84.211 | 147697 | 147752 | 71682 | 71738 | 1.68E-06 |
| chr1 | chr1 | F | 50 | 86 | 200891 | 200940 | 75146 | 75195 | 1.68E-06 |
| chr1 | chr1 | P | 29 | 100 | 196513 | 196541 | 79078 | 79106 | 1.68E-06 |
| chr1 | chr1 | F | 33 | 96.97 | 187566 | 187598 | 82960 | 82991 | 1.68E-06 |
| chr1 | chr1 | F | 35 | 94.286 | 151550 | 151584 | 83623 | 83657 | 1.68E-06 |
| chr1 | chr1 | P | 41 | 90.244 | 194372 | 194412 | 104376 | 104416 | 1.68E-06 |
| chr1 | chr1 | F | 29 | 100 | 159104 | 159132 | 115959 | 115987 | 1.68E-06 |
| chr1 | chr1 | P | 38 | 92.105 | 6205 | 6242 | 147636 | 147673 | 1.68E-06 |
| chr1 | chr1 | P | 57 | 84.211 | 199384 | 199440 | 156616 | 156671 | 1.68E-06 |
| chr1 | chr1 | P | 48 | 87.5 | 196156 | 196201 | 157864 | 157911 | 1.68E-06 |
| chr1 | chr1 | P | 38 | 92.105 | 104379 | 104416 | 194372 | 194409 | 1.68E-06 |
| chr1 | chr1 | F | 28 | 100 | 79078 | 79105 | 319 | 346 | 6.05E-06 |
| chr1 | chr1 | P | 31 | 96.774 | 195544 | 195574 | 21820 | 21850 | 6.05E-06 |
| chr1 | chr1 | P | 37 | 91.892 | 196516 | 196552 | 23225 | 23261 | 6.05E-06 |
| chr1 | chr1 | P | 41 | 90.244 | 28241 | 28279 | 23546 | 23586 | 6.05E-06 |
| chr1 | chr1 | P | 34 | 94.118 | 24051 | 24084 | 24051 | 24084 | 6.05E-06 |
| chr1 | chr1 | P | 31 | 96.774 | 95327 | 95357 | 25344 | 25374 | 6.05E-06 |
| chr1 | chr1 | P | 28 | 100 | 69082 | 69109 | 28140 | 28167 | 6.05E-06 |
| chr1 | chr1 | P | 28 | 100 | 69082 | 69109 | 28169 | 28196 | 6.05E-06 |
| chr1 | chr1 | P | 28 | 100 | 79078 | 79105 | 28260 | 28287 | 6.05E-06 |
| chr1 | chr1 | P | 28 | 100 | 69082 | 69109 | 28260 | 28287 | 6.05E-06 |
| chr1 | chr1 | F | 31 | 96.774 | 147643 | 147673 | 65599 | 65629 | 6.05E-06 |
| chr1 | chr1 | P | 28 | 100 | 196514 | 196541 | 69082 | 69109 | 6.05E-06 |
| chr1 | chr1 | F | 31 | 96.774 | 102415 | 102445 | 74598 | 74628 | 6.05E-06 |
| chr1 | chr1 | F | 28 | 100 | 187571 | 187598 | 79075 | 79102 | 6.05E-06 |
| chr1 | chr1 | F | 28 | 100 | 82967 | 82994 | 79078 | 79105 | 6.05E-06 |
| chr1 | chr1 | P | 34 | 94.118 | 177621 | 177654 | 87670 | 87703 | 6.05E-06 |
| chr1 | chr1 | P | 48 | 87.5 | 195904 | 195949 | 156620 | 156665 | 6.05E-06 |
| chr1 | chr1 | P | 31 | 96.774 | 180080 | 180110 | 167962 | 167992 | 6.05E-06 |
| chr1 | chr1 | P | 34 | 94.118 | 87673 | 87706 | 177618 | 177651 | 6.05E-06 |

P, Palindromic repeat, F, forward repeat.

Table S8 The nucleotide variability of B. rapa var. Purpuraria mt genome

| No. | Region | Pi | Total Number of mutations | Length |
| --- | --- | --- | --- | --- |
| 1 | *gene1.ccmC* | 0.04585 | 84 | 756 |
| 2 | *gene10.nad4L* | 0.02355 | 14 | 303 |
| 3 | *gene11.cox1* | 0.03196 | 116 | 1695 |
| 4 | *gene12.rps3* | 0.06481 | 252 | 1713 |
| 5 | *gene13.rpl16* | 0.03691 | 24 | 558 |
| 6 | *gene14.rpl5* | 0.04806 | 69 | 564 |
| 7 | *gene15.rps14* | 0.0198 | 12 | 303 |
| 8 | *gene16.cob* | 0.03325 | 104 | 1305 |
| 9 | *gene17.cox2* | 0.06892 | 127 | 1431 |
| 10 | *gene18.ccmFc* | 0.05802 | 205 | 1716 |
| 11 | *gene19.cox3* | 0.03187 | 63 | 798 |
| 12 | *gene2.nad2* | 0.0215 | 74 | 1467 |
| 13 | *gene20.ccmFn* | 0.05687 | 154 | 1797 |
| 14 | *gene21.atp9* | 0.0709 | 42 | 300 |
| 15 | *gene22.nad3* | 0.04575 | 35 | 357 |
| 16 | *gene23.rps12* | 0.04263 | 34 | 378 |
| 17 | *gene24.ccmB* | 0.03236 | 54 | 621 |
| 18 | *gene25.nad1* | 0.02618 | 56 | 978 |
| 19 | *gene26.nad6* | 0.03167 | 53 | 654 |
| 20 | *gene27.nad4* | 0.03011 | 109 | 1488 |
| 21 | *gene28.mttB* | 0.02625 | 24 | 862 |
| 22 | *gene29.rpl2* | 0.07182 | 140 | 1065 |
| 23 | *gene3.rpl10* | 0.14222 | 48 | 489 |
| 24 | *gene30.atp1* | 0.04699 | 174 | 1536 |
| 25 | *gene31.rrn26* | 0.02046 | 127 | 3775 |
| 26 | *gene32.rps7* | 0.0179 | 16 | 447 |
| 27 | *gene33.matR* | 0.05357 | 265 | 2094 |
| 28 | *gene34.nad5* | 0.02098 | 101 | 2016 |
| 29 | *gene35.rrn18* | 0.04281 | 162 | 2026 |
| 30 | *gene36.rrn5* | 0.01905 | 5 | 121 |
| 31 | *gene4.rps4* | 0.07436 | 185 | 1106 |
| 32 | *gene5.nad7* | 0.0213 | 67 | 1185 |
| 33 | *gene6.atp8* | 0.07195 | 75 | 495 |
| 34 | *gene7.nad9* | 0.02119 | 35 | 573 |
| 35 | *gene8.atp6* | 0.05245 | 87 | 1347 |
| 36 | *gene9.atp4* | 0.0659 | 86 | 597 |

Table S9 Homology analysis of the mt and cp genomes of *B. rapa* var. *Purpuraria*

| Genome | Genome length(bp) | Homologous sequence length(bp) | Proportion(%) |
| --- | --- | --- | --- |
| Cp | 153483 | 13153 | 8.57 |
| Mt | 219775 | 8961 | 4.08 |


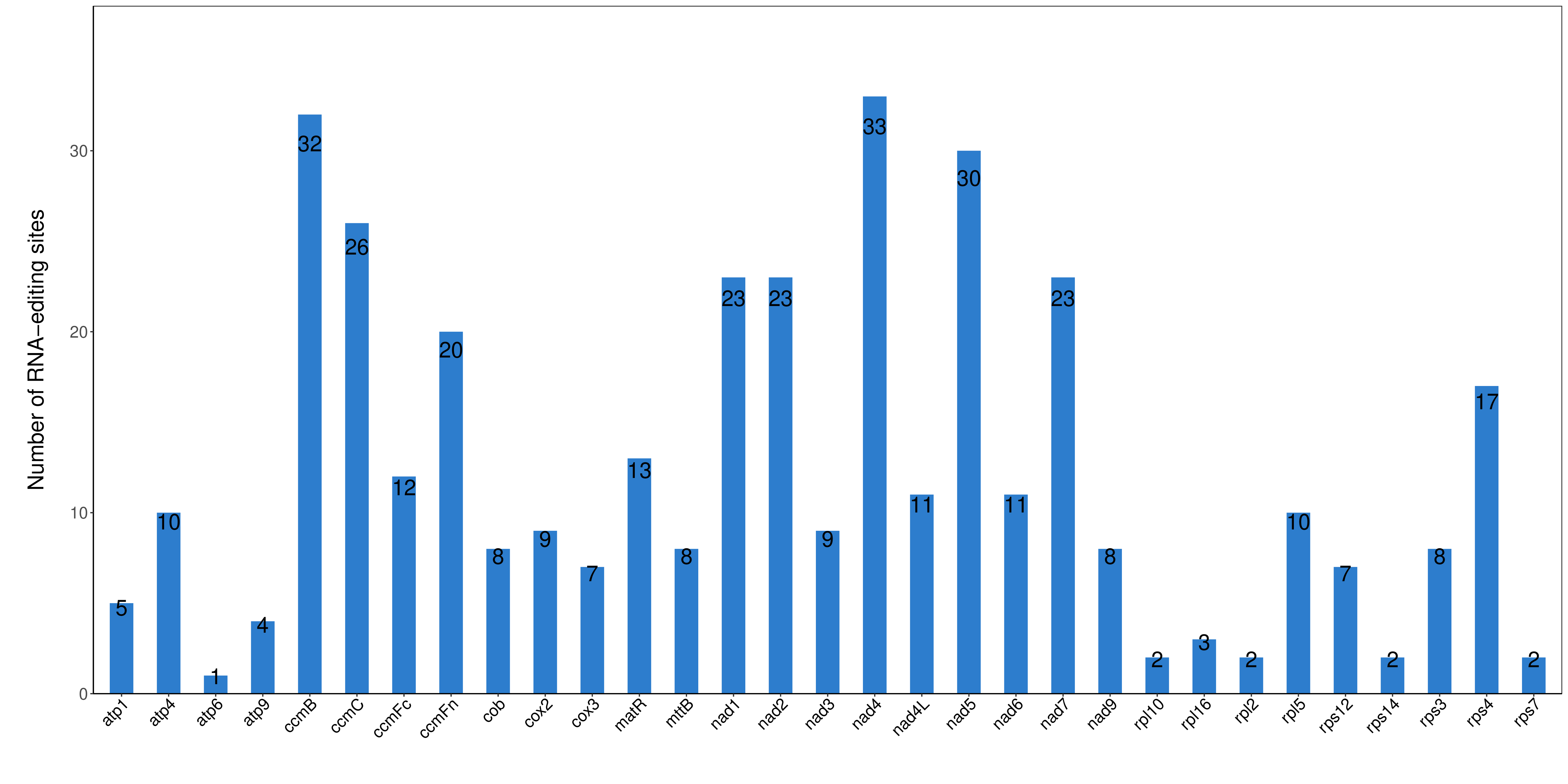


Figure S1 The distribution of RNA-editing sites of each PCG in the *B. rapa* var. *Purpuraria* mt genome.
